# In-depth Phylogenomic Analysis of Arbuscular Mycorrhizal Fungi Based on a Comprehensive Set of *de novo* Genome Assemblies

**DOI:** 10.3389/ffunb.2021.716385

**Published:** 2021-09-29

**Authors:** Merce Montoliu-Nerin, Marisol Sánchez-García, Claudia Bergin, Verena Esther Kutschera, Hanna Johannesson, James D. Bever, Anna Rosling

**Affiliations:** ^1^Department of Ecology and Genetics, Evolutionary Biology, Uppsala University, Uppsala, Sweden; ^2^Department of Forest Mycology and Plant Pathology, Uppsala Biocentre, Swedish University of Agricultural Sciences, Uppsala, Sweden; ^3^Microbial Single Cell Genomics Facility, Department of Cell and Molecular Biology, Science for Life Laboratory, Uppsala University, Uppsala, Sweden; ^4^Department of Biochemistry and Biophysics, National Bioinformatics Infrastructure Sweden, Science for Life Laboratory, Stockholm University, Solna, Sweden; ^5^Department of Organismal Biology, Systematic Biology, Uppsala University, Uppsala, Sweden; ^6^Department of Ecology and Evolutionary Biology, and Kansas Biological Survey, University of Kansas, Lawrence, KS, United States

**Keywords:** genomics, phylogenetic, single nuclei sequencing, topology, Glomeromycota

## Abstract

Morphological characters and nuclear ribosomal DNA (rDNA) phylogenies have so far been the basis of the current classifications of arbuscular mycorrhizal (AM) fungi. Improved understanding of the evolutionary history of AM fungi requires extensive ortholog sampling and analyses of genome and transcriptome data from a wide range of taxa. To circumvent the need for axenic culturing of AM fungi we gathered and combined genomic data from single nuclei to generate *de novo* genome assemblies covering seven families of AM fungi. We successfully sequenced the genomes of 15 AM fungal species for which genome data was not previously available. Comparative analysis of the previously published *Rhizophagus irregularis* DAOM197198 assembly confirm that our novel workflow generates genome assemblies suitable for phylogenomic analysis. Predicted genes of our assemblies, together with published protein sequences of AM fungi and their sister clades, were used for phylogenomic analyses. We evaluated the phylogenetic placement of Glomeromycota in relation to its sister phyla (Mucoromycota and Mortierellomycota), and found no support to reject a polytomy. Finally, we explored the phylogenetic relationships within Glomeromycota. Our results support family level classification from previous phylogenetic studies, and the polyphyly of the order Glomerales with Claroideoglomeraceae as the sister group to Glomeraceae and Diversisporales.

## Introduction

Arbuscular mycorrhizal (AM) fungi are an ecologically important group of fungi that form ubiquitous associations with plants, establishing symbiosis with up to 80% of land plant species (Parniske, 2008; Smith and Read, 2010). Arbuscular mycorrhizal fungi play foundational roles in terrestrial productivity, and there is accumulating evidence that AM fungal taxa are functionally distinct and that their community composition have functional consequences for terrestrial ecosystems (Hoeksema et al., 2018; Koziol et al., 2018). Therefore, progress in understanding the ecologically distinct roles of AM fungi depends upon accurate phylogenetic inference at all taxonomic levels.

Available literature identify that all AM fungi form a monophyletic lineage within the fungal kingdom. This lineage is taxonomically classified either as a phylum, Glomeromycota (Schüβler et al., 2001; Hibbett et al., 2007; Schüßler and Walker, 2010; Tedersoo et al., 2018), or as the sub-phylum Glomeromycotina, which together with Mortierellomycotina and Mucoromycotina, make up the phylum Mucoromycota (Spatafora et al., 2016; James et al., 2020; Li et al., 2021). The current consensus classification of AM-fungal species into genera and families was established by Redecker et al. in 2013, when systematists with long experience in the biology and taxonomy of AM fungi joined forces to integrate morphological and molecular phylogenetic information to generate a meaningful classification that reflects evolutionary relationships (Redecker et al., 2013). Molecular data at the time was primarily based on partial sequences of nuclear ribosomal DNA (rDNA). Around 300 species of AM fungi are currently described and classified into 33 genera, twelve families and four orders (Redecker et al., 2013; Wijayawardene et al., 2018).

Molecular identification of AM fungi to genera and family is usually possible based on sequences of small subunit (SSU) or large subunit (LSU) regions of rDNA genes (Redecker et al., 2013; Öpik et al., 2014). However, species level inference based on rDNA genes is difficult due to high levels of intra species variation (Stockinger et al., 2009; House et al., 2016). While the rDNA operon is commonly found in a multi-copy tandem repeat organization across fungi (Lofgren et al., 2019), in AM fungi different rDNA variants can be scattered across the genome (VanKuren et al., 2013; Maeda et al., 2018) and lack the usual tandem organization (Maeda et al., 2018). The fact that rDNA genes are present as paralogs in AM fungal genomes likely explains the high levels of within strain and species diversity of rDNA sequences.

Limitations of single-locus phylogenetic inference and paralogous nature of rDNA genes in AM fungi calls for the need to generate extensive ortholog datasets from taxa representing different families, in order to accurately infer phylogenetic relationships among AM fungal lineages. One approach in this direction was achieved in a recent study using spore transcriptomic data for phylogenomic analysis of nine taxa from seven families (Beaudet et al., 2018). In this study, Glomerales was recovered as polyphyletic, in contrast to earlier rDNA phylogenies where Glomerales was found to be monophyletic (Krüger et al., 2012). Other phylogenomic studies have not adressed relations among families largely due to limited taxon sampling (Morin et al., 2019; Sun et al., 2019; Venice et al., 2020; Li et al., 2021). Due to difficulties in obtaining enough pure DNA for whole genome sequencing, available genomic data still represent only a fraction of the diversity of AM fungi.

Progress in AM fungal genomics has been limited by their biology. Arbuscular mycorrhizal fungi complete their life cycle underground, as obligate symbionts of plant roots, and reproduce through multinuclear asexual spores (Bonfante and Genre, 2010). The spores are the largest isolable structure produced, but large-scale isolation of spores is needed in order to obtain enough DNA for whole genome sequencing. Such large-scale harvest of spores is only possible with AM fungi grown in axenic cultures where the fungus produces spores in a compartment separate from the transformed plant roots that it associates with (Tisserant et al., 2013), or for the rare taxa, such as *Diversispora epigaea* that forms fruitbodies above ground and from which large amounts of spores can be extracted (Sun et al., 2019). Axenic culturing methods are time-consuming and have only been successful for a handful of species (Kameoka et al., 2019). Due to the difficulty of producing clean cultures and isolate high quality DNA extracts for the majority of AM fungal species, it has been a slow path toward genomic studies of AM fungi (Tisserant et al., 2013; Lin et al., 2014; Beaudet et al., 2018; Kobayashi et al., 2018; Morin et al., 2019; Sun et al., 2019; Venice et al., 2020).

AM fungal hyphae and their asexual spores are coenocytic, and sequence analysis of individual nuclei have been used to analyze intra organismal polymorphism mainly by mapping reads of single nuclei to reference genomes of the model AM fungus *Rhizophagus irregularis* (Lin et al., 2014; Ropars et al., 2016; Chen et al., 2018). To circumvent the obstacle of pure culturing, we recently presented a workflow that takes advantage of automated nuclei sorting by extracting nuclei from un-germinated spores, directly extracted from soil (Montoliu-Nerin et al., 2020). Single nuclei of *Claroideoglomus claroideum* were sorted, followed by whole genome amplification (WGA) and sequencing. Finally, the data from several nuclei were combined to build a *de novo* genome assembly. With this novel workflow AM fungal genome assemblies can be obtained from as little as one single spore, independently of the species ability to grow in axenic cultures (Montoliu-Nerin et al., 2020). Similar approaches have been successfully applied in other organisms for which limited access to pure biological material suitable for extraction of high-quality DNA has prevented genome sequencing (Stepanauskas and Sieracki, 2007; Woyke et al., 2009; Heywood et al., 2011; Yoon et al., 2011; Walker et al., 2014; Wideman et al., 2019).

In this study, we sorted and sequenced nuclei from AM fungal spores representing species across Glomeromycota, aiming to obtain genomic information from two taxa for each genus. To evaluate the quality of assemblies generated by our workflow, we included *Rh. irregularis* DAOM197198, a strain for which a reference genome generated from an axenic culture is available (Chen et al., 2018), and compared this published assembly with our newly generated assembly. A final count of 21 strains, from 12 genera, across seven families were successfully sequenced, and *de novo* genome assemblies were constructed. Our dataset includes 15 species for which genome data was not previously available. This comprehensive taxon sampling allowed us to infer evolutionary relationships among AM fungi. Furthermore, the release of new whole genome assemblies provides a resource to the research community, for those interested in further exploring genetics and evolution of this important group of fungi.

## Materials and Methods

### Fungal Strains

Taxa were initially selected to represent 15 genera across eight families in Glomeromycota (Schüßler and Walker, 2010; Redecker et al., 2013), aiming for two species per genus (Supplementary Table 1). The isolates were obtained as whole inoculum from the International culture collection of (vesicular) arbuscular mycorrhizal fungi (INVAM) at West Virginia University, Morgantown, WV, USA, or James D. Bever's lab, University of Kansas, USA, with the exception of *Rh. irregularis* DAOM197198 which was obtained as a tube of spores from Agriculture and Agri-food Canada, Government of Canada. In addition to the AM fungi sampled for this study, we included published annotated genome- and transcriptome assemblies of AM fungi (Glomeromycota; 13 taxa, Supplementary Table 2). Furthermore, we included all species of the closest sister lineages with available genome assemblies and annotations (December 2019) in the JGI (Joint Genome Institute) database, i.e. Mortierellomycota (2 taxa, Mondo et al., 2017a; Uehling et al., 2017) and Mucoromycota (12 taxa, Ma et al., 2009; Wang et al., 2013; Schwartze et al., 2014; Chibucos et al., 2016; Corrochano et al., 2016; Mondo et al., 2017a,b; Chang et al., 2019) (Supplementary Table 2). Finally, members of Dikarya were included as outgroup, with three representatives of Ascomycota, one taxon each from the subphyla Taphrinomycotina (Pomraning et al., 2018), Saccharomycotina (Wood et al., 2002), and Pezizomycotina (Martin et al., 2010); and three representatives of Basidiomycota, one taxon each from the subphyla Agaricomycotina (Martin et al., 2008), Ustilaginomycotina (Kämper et al., 2006), and Pucciniomycotina (Schwessinger et al., 2018) (Supplementary Table 2).

### Nuclear Sorting and Whole Genome Amplification

Spores were extracted from whole inoculum cultures by sieving, followed by a sucrose gradient centrifugation as described in Montoliu-Nerin et al. (2020). A single spore or a pool of spores (Supplementary Table 1) were then rinsed and stored in 20 μl ddH_2_O in a 1.5 ml tube. After adding 180 μl of 1 × PBS spores were crushed with a sterile pestle and DNA was stained by adding 1 μl of 200x SYBR Green I Nucleic Acid stain (Invitrogen™, Thermo Fisher Scientific, MA, USA). Sorting of the nuclei proved to be more successful when the crushed spore solution was transferred to the small 0.5 ml tube for staining. This allowed the spore debris to settle while the nuclei remained in solution. The sample was left staining for 30–60 min, and lower sorting performance was observed when exceeding that time. The nuclear sorting was performed at the SciLifeLab Microbial Single Cell Genomics Facility with a MoFlo™ Astrios EQ sorter (Beckman Coulter, USA), as in Montoliu-Nerin et al. (2020). Briefly, a 100 μm nozzle was used and the sheath fluid, 0.1 μm filtered 1x PBS, was run at 25 psi. Nuclei populations were identified via nuclei acid staining using the 488 nm laser and a 530/40 nm bandpass filter over forward or side scatter. Individual nuclei were deposited into 96- or 384-well plates using stringent single-cell sort settings (single mode, drop envelope 1). These sort-settings abort target cells if another particle of any type is in the same or the neighboring drop, thereby increasing the number of aborts while ensuring that only one particle gets sorted per well. Each day of sorting, the sort precision was determined with beads sorted onto a slide and counted manually under the microscope. A low event rate was used to decrease the risk of sorting doublets, for most samples below 500 events per second with a drop generation of >40,000 per second corresponding to well-below 1% of nuclei in the samples. Most of the remaining particles were low in SYBR Green fluorescence. To each plate, 48 wells were used for sorting single nuclei or up to four pools of five nuclei, leaving the rest of the wells empty. Plates with sorted nuclei were stored at −80°C.

DNA from the nuclei samples was amplified with the enzyme Phi29 via multiple displacement amplification (MDA) under clean (i.e., amplicon and contaminant free) conditions using the RepliPhi kit (Epicenter) in a 15 μl reaction volume in 96-well plates or with the Repli-g Single Cell kit (Qiagen) in a 10 μl reaction volume in 384-well plates. The nucleic acid stain SYTO 13 was added to the reaction to follow the DNA amplification over time. Protocol including plate size and MDA kit was changed over time (Supplementary Table 1).

### Sequencing of Amplified Nuclei Samples

We screened MDA nuclei samples by PCR amplification of rDNA markers using fungal and bacterial specific primers, following the protocol in Montoliu-Nerin et al. (2020). Multiple displacement amplification nuclei samples that scored positive for fungi and negative for bacteria were selected for sequencing. For samples with enough DNA the TruSeq PCRfree DNA library preparation kit (Illumina Inc.) was used. In total, 7–24 nuclei from each isolate were independently sequenced with Illumina HiSeq-X, at the SNPandSEQ Technology Platform in Uppsala at the National Genomics Infrastructure (NGI) Sweden and Science for Life Laboratory, as in Montoliu-Nerin et al. (2020). Detailed information on sorting, MDA results, PCR screening, and selection of nuclei samples is available in Supplementary Data File 1.

### Genome Assembly and Strain Verification

Whole genome assembly was performed according to assembly workflow 3 as described in Montoliu-Nerin et al. (2020), in which all sets of reads from individually sequenced nuclei samples from each strain were combined and normalized using bbnorm of BBMap v.38.08 (Bushnell, 2016), setting an average depth of 100X, and then assembled using SPAdes v.3.12.0 (Bankevich et al., 2012). We chose this workflow for the current study as it gives a good representation and accuracy of single copy genes, making it more suitable for downstream phylogenomic analyses than the other two workflows developed (Montoliu-Nerin et al., 2020). We used Quast v.4.5.4 (Gurevich et al., 2013) to quantitatively assess the assemblies (Supplementary Table 3) and ran BUSCO v.3.0.2b (Simão et al., 2015) to assess completeness of the genome, using fungi_odb9 as lineage setting, and rhizopus_oryzae as species set (Supplementary Tables 3, 4). Raw reads and *de novo* genome assemblies are deposited in ENA in the project PRJEB45340.

To verify strain identity based on a reconstructed ribosomal gene phylogeny, we extracted the ribosomal gene operon from each newly assembled genome. For strains in the family Claroideoglomeraceae only one of its highly diverging rDNA sequences (VanKuren et al., 2013) was retrieved as a complete operon. In earlier work, when the genome of *C. claroideum* was assembled by combining single nuclei assemblies, we could identified both rDNA variants, but not when the genome assembly was generated from combined and normalized reads (Montoliu-Nerin et al., 2020). The SSU region was combined with the taxon rich SSU alignment from Krüger et al. (2012). The whole rDNA operons extracted from genome assemblies with verified identity, were aligned and a phylogeny was reconstructed with RAxML v.8.2.10 (Stamatakis, 2014), implementing the GTR model and with IQ-TREE v.1.6.5 (Nguyen et al., 2015), using ModelFinder (Kalyaanamoorthy et al., 2017) and searching for the best partitioning scheme. We ran both analyses with 1,000 bootstrap replicates. Extracted rDNA operons for all *de novo* genome assemblies available in the linked public OSF repository.

### Genome Annotation

Each genome assembly was annotated using a snakemake workflow (Köster and Rahmann, 2012) v.2.0. The workflow is publicly available at https://bitbucket.org/scilifelab-lts/genemark_fungal_annotation/ (tag v.3.0, with minor updates providing the same functionality). Briefly, repeats and transposable elements were *de novo* predicted in each of the assemblies using RepeatModeler v.1.0.8 (Smit and Hubley, 2008) and the resulting repeat library was used to mask each genome assembly using RepeatMasker v.4.0.7 (Smit et al., 2015). UniProt/Swiss-Prot (Consortium, 2018) protein sequences (downloaded 8 May 2018) were aligned to each of the repeat-masked genome assemblies with MAKER v.3.01.1-beta (Cantarel et al., 2008). Protein coding genes were *de novo* predicted from each of the repeat-masked genome assemblies with GeneMark-ES v.4.33 (Ter-Hovhannisyan et al., 2008), providing the genomic locations of Uniprot/Swiss-Prot proteins aligned to the genome assembly to guide the gene predictions. Minimum contig size to be included in self-training of the GeneMark gene prediction algorithm was calculated to include at least 10 Mb of training data, depending on the level of fragmentation of the assembly, and was set accordingly using the parameter “–min_contig” (Table of specific parameter used for each assembly is available in the linked public OSF). Protein and gene names were assigned to the gene predictions using a BLASTp v.2.7.1 (Camacho et al., 2009) search of predicted protein sequences against the UniProt/Swiss-Prot database with default e-value parameters (1 × 10^−5^). InterProScan v.5.30-69.0 (Cock et al., 2013) was used to collect predictive information about the predicted proteins' functions.

### Assessing Assembly Quality Using *Rhizophagus irregularis* DAOM197198

To confirm the accuracy of assemblies generated in our workflow we included the reference strain *Rh. irregularis* DAOM197198 (Supplementary Table 1) and compared our *de novo* genome assembly to a published high-quality genome assembly DAOM197198 v.2.0 (Supplementary Table 2) (Chen et al., 2018). Including this well-characterized strain allowed us to assess the performance of our assembly workflow. To assess efficiency and coverage of single nuclei MDA and sequencing, we mapped reads from individual nuclei against the published reference assembly and to our *de novo* assembly of *Rh. irregularis* DAOM197198, using BWA 0.7.15 (Li and Durbin, 2009), and measured both % of reads mapping and % of assembly covered with mapped reads using Qualimap 2.2.1 (Okonechnikov et al., 2016) and bamtools v.2.3.0 stats (Barnett et al., 2011). We also tested for polymorphism introduced during MDA by pair-wise alignment of the 271 BUSCO genes retrieved from both assemblies using MAFFT v.7.407 (Katoh and Standley, 2013). Percentage similarity for the alignments was calculated with esl-alistat in HMMer v.3.2.1 (Hancock and Bishop, 2004). Finally, to take advantage of the ready-made comparative analysis of OrthoFinder v.2.4.0 (Emms and Kelly, 2018), we used this software (with standard settings) to identify orthogroups in the two genome assemblies.

### Phylogenomic Analyses

Phylogenomic analyses were performed at different taxonomic scales, using six datasets with different taxon sampling (Supplementary Table 5). The first dataset was designed to explore the relationship of Glomeromycota with its sister phyla Mucoromycota and Mortierellomycota, and included genome data from Dikarya as outgroup (Supplementary Tables 2, 3). Two more datasets were designed to explore the relationships within Glomeromycota, including as many AM fungal taxa as possible, as well as members of its sister phyla. One of them included assembled transcriptomic data for Glomeromycota (Beaudet et al., 2018), while the other included only genomic data. For each of these two latter datasets, single copy orthologs (SCOs) were identified from the gene predictions using OrthoMCL v.2.0.9 (Li et al., 2003) with default parameters, requiring that SCOs were present in >50% of the taxa. Three additional datasets were designed to further explore conflicting topologies within Glomeromycota. Two of the three, were taxon-rich, including all species in Glomeromycota with available genome data (27 taxa), for one we retrieved SCOs present in >50% of the taxa, while for the other we retrieved SCOs present in all 27 taxa. The last of the three Glomeromycota datasets was designed to evaluate whether the inclusion of assemblies with low BUSCO completeness (<80%) impacts the phylogenetic reconstruction. Thus, this dataset included at least one species from each available genus with >90% estimated BUSCO completeness, except for *Ra. fulgida* and *Ac. colombiana*, for which BUSCO completeness was estimated to 82 and 87%, respectively (Supplementary Tables 3, 4). Single copy orthologs that were present in all 15 taxa were included in this dataset (Supplementary Table 5).

Amino acid sequences were aligned using MAFFT v.7.407 (Katoh and Standley, 2013). Poorly aligned regions were removed using trimAl v.1.4.1 (Capella-Gutiérrez et al., 2009) with a gap threshold of 0.1 (0.2 in the dataset with only 15 taxa selected, Supplementary Table 4). Individual SCO alignments were removed if shorter than 100 amino acids. Single copy ortholog alignments were used either separately, to produce individual gene trees, or concatenated, to produce best maximum likelihood (ML) trees. Individual SCO alignments were concatenated into a supermatrix using the script geneStitcher.py (Schluter, 2016), which also produces a gene partition file. Lists of SCOs used for phylogenomic inferences from each dataset and their corresponding gene annotations are available in the linked public OSF repository.

Phylogenetic inferences based on the concatenated sets of SCOs were performed using two ML methods. First, ML phylogenies were inferred using RAxML v.8.2.10 (Stamatakis, 2014), with 100 bootstrap replicates, and with a partitioned model that treated each SCO as a separate partition and implementing the PROTOGAMMAWAG model for all partitions. Secondly, ModelFinder (Kalyaanamoorthy et al., 2017) was run for every partition, and a best ML tree was generated with 100 bootstrap replicates in IQ-TREE v.1.6.5 (Nguyen et al., 2015). Topologies and support values from both ML inference methods were highly comparable, therefore, we only present the RAxML topologies but adding support values from the IQ-TREE analysis. Phylogenetic inferences were also performed with ASTRAL-III v.5.7.3 (Zhang et al., 2018), a method consistent with a multi-species coalescent model. For this, we used individual gene trees inferred with IQ-TREE using the automated detection for the best-fitting model (-MFP) and 100 bootstrap replicates. The topological robustness was evaluated with a multi-locus bootstrapping (MLBS) and local posterior probabilities (LPP). For the dataset including Glomeromycota and its sister phyla, a Bayesian phylogeny was inferred using Phylobayes (Lartillot et al., 2009), under the site-heterogeneous CAT+GTR+G4 model on a total alignment of 144,177 amino acids. Two chains were run and convergence was evaluated using the commands tracecomp and bpcomp in Phylobayes, which was achieved after 120,000 and 200,000 generations.

We evaluated the phylogenetic placement of Glomeromycota in relation to Mucoromycota and Mortierellomycota, and the relationships within Glomeromycota, specifically Claroideoglomeraceae, Glomeraceae and Diversisporales. For this, we examined the support among individual gene trees for alternative branching orders. We performed a polytomy test in ASTRAL-III v.5.7.3 to identify evidence for hard polytomies. The test uses quartet gene tree frequencies to evaluate whether a polytomy could be rejected (Sayyari and Mirarab, 2018).

To further explore the relationships among lineages of AM fungi, splits networks were produced for two datasets (Supplementary Table 5) using IQ-TREE v.1.6.5 (Nguyen et al., 2015) with the command iqtree –net. Networks were visualized in SplitsTree5 (Huson and Bryant, 2006) with a maximum dimension of 2. For the dataset with 15 selected AM fungal taxa, topologies branching over the tree landscape were also visualized and the consensus topologies were analyzed using DensiTree v.2.01 (Bouckaert and Heled, 2014) based on the previously inferred individual gene trees from IQ-TREE.

## Results

### Presenting 21 *de novo* Genome Assemblies of AM Fungi

Using our novel workflow for *de novo* assembly of genomes by combining single nuclei sequence data (Montoliu-Nerin et al., 2020), we aimed to sequence 31 AM fungal isolates, with at least two taxa from each 15 genera across eight families (Supplementary Table 1). Spores from all 31 isolates were extracted for nuclei sorting and DNA amplification. For two of the taxa, *Archaeospora trappei* and *Entrophospora infrequence*, we failed to sort nuclei, and these were thus omitted from subsequent methods. After WGA with MDA on the sorted nuclei, samples from the remaining 29 isolates were screened by PCR amplification of the rDNA barcode region for presence of DNA of fungal and bacterial origin. Presence of fungal DNA was confirmed for 25 of the isolates, while samples from four isolates did not amplify the fungal rDNA barcode region and were thus excluded from sequencing (Supplementary Table 1). Genome assemblies of the 25 isolates ranged from 50 to 493 Mb in size, with numbers of gene predictions ranging from 11,400 to 46,500 and BUSCO completeness between 55 and 95% (Supplementary Tables 3, 4). Four of these assemblies were later removed due to misidentification of strains, see Isolate Identification in rDNA-based Phylogeny below, resulting in a final number of 21 genomes presented and used in the phylogenomic analyses.

Based on the comparison of *Rh. irregularis* DAOM197198 genome assemblies, we found that single nuclei MDA and sequencing were highly accurate and efficient in our workflow. On average, around 99% of the reads mapped to both our *de novo* genome assembly and the published reference genome v.2.0 of *Rh. irregularis* DAOM197198 (Supplementary Table 6). Reads from individual nuclei covered on average 50% of both assemblies and when combined the reads covered close to 95% of the reference genome v.2.0 (Supplementary Table 6). Together these results demonstrate that reads from single amplified and sequenced nuclei fully match the published reference and that the whole genome is represented among the reads. Pair-wise alignment of the 271 BUSCO genes retrieved in both assemblies of *Rh. irregularis* DAOM197198 demonstrate high consistency with an average similarity of 99.7% across nucleotide alignments. Of the 271 pairwise aligned BUSCO genes, a total of 260 were identical between the two assemblies, corresponding to 96% of the retrieved BUSCO genes. However, only 60% similarity was detected in one of the 271 pairwise alignments, and ten alignments ranged in similarity between 84 and 99% (Supplementary Data File 2). High similarity in pairwise alignments of BUSCO genes retrieved from the two assemblies demonstrates that random errors possibly introduced during MDA are not retained to a large extent in genes in the assembled genome when reads from single nuclei are combined and normalized before assembling with SPAdes. In our assembly of *Rh. irregularis* DAOM197198, 23,258 genes were predicted (Supplementary Table 3) compared to 26,183 genes predicted in the published assembly of *Rh. irregularis* DAOM197198 v.2.0 (Chen et al., 2018). We demonstrate that our *de novo* genome assembly for *Rh. irregularis* DAOM197198 contained a largely overlapping set of genes in orthogroups present in the published *Rh. irregularis* DAOM197198 reference genome v.2.0. Across the two genome assemblies of the same strain, a total of 13,908 orthogroups were identified including 88% of all predicted genes across the two assemblies, of these, 94% were shared between the two genome assemblies (Supplementary Table 7). Interestingly, both genome assemblies contain orthogroups not recovered in the other, 403 unique to v.2.0 and 380 unique to our *de novo* assembly (Supplementary Table 7).

### Isolate Identification in rDNA-based Phylogeny

The complete rDNA operon, including SSU, ITS1, 5,8s, ITS2, and LSU regions was extracted from the 25 newly generated genome assemblies. To confirm genus level identity of the 25 isolates for which we generated genome assemblies in this study, the SSU rDNA region was extracted and placed into the taxon-rich Glomeromycota phylogeny of Krüger et al. (2012) (Supplementary Figure 1). For five isolates, the species name did not correspond with the phylogenetic placement, revealing that these isolates were originally misidentified. Four of these were removed. First, the isolate *Rhizophagus intraradices* FL208A clustered within the genus *Funneliformis* (Supplementary Figure 1), more specifically, together with samples of *Funneliformis mosseae*. Morphological examination of this strain was consistent with its original identification as *Rh. intraradices*. We could not verify that spores with the correct morphology had been extracted for nuclei sorting. Therefore, this genome assembly was excluded from further analyses. The isolate *Funneliformis caledonius* UK204 also clustered with samples representing *F. mosseae* (Supplementary Figure 1), but since the genus placement was correct the strain was kept as *F. caledonius* for further analysis. The isolate *Di. epigaea* AZ150B was phylogenetically misplaced based on its rDNA SSU sequence (Supplementary Figure 1), and the assembly had the highest GC content among our assemblies (Supplementary Table 3), probably due to bacterial contamination. The MDA success for this strain was low and this was the only strain for which we included two nuclei samples, out of seven sequenced, where PCR had indicated the presence of bacterial DNA. We thus decided to discard this assembly since a genome of *Di. epigaea* is publicly available (Sun et al., 2019) and was included in the analyses. Finally, the isolates *Archaeospora scheckii* CL383 and *Septoglomus viscosum* MD215 were placed in the family Paraglomeraceae (Supplementary Figure 1), and subsequently eliminated from further analyses, as two *Paraglomus* isolates were already included. After this confirmation step, *de novo* genome assemblies representing 21 isolates were kept for the phylogenomic analyses. A phylogenetic analysis of the entire extracted rDNA operon from the 21 genome assemblies, as well as that of *C. claroideum* previously generated in our group, showed that, in line with earlier phylogenetic results based on rDNA genes (Redecker et al., 2013), Glomerales is monophyletic, albeit with bootstrap support (BS) of just over 80% (Supplementary Figure 2).

### Phylogenomic Analysis of Glomeromycota

To place Glomeromycota in relation to its sister phyla, phylogenetic trees were built from a dataset that included members of Glomeromycota, Mucoromycota and Mortierellomycota, and Dikarya as outgroup, with 178 SCOs that were shared among >50% of the taxa. The concatenated alignment had a length of 76,737 amino acids. In the RAxML phylogeny, Glomeromycota and Mortierellomycota form a monophyletic clade (80% BS), with Mucoromycota as their sister group (100% BS) (Supplementary Figures 3, 4A). However, in the IQ-TREE phylogeny, Mortierellomycota and Mucoromycota form a monophyletic clade (43% BS), sister to Glomeromycota (100% BS) (Supplementary Figure 4B). The ASTRAL analysis recovered the same topology as the RAxML analysis, but with low support (LPP = 0.53) (Supplementary Figure 4C). The quartet gene tree frequencies were very similar for the three alternative topologies (q1 = 0.37, q2 = 0.29, q3 = 0.34), suggesting that a polytomy cannot be rejected (*p*-value = 0.71) (Supplementary Figure 5). The relationships among the three sister phyla remain unresolved in our analysis, likely because of the incomplete taxon sampling, in particular for Mortierellomycota that was only represented by two species.

**Figure 1:**
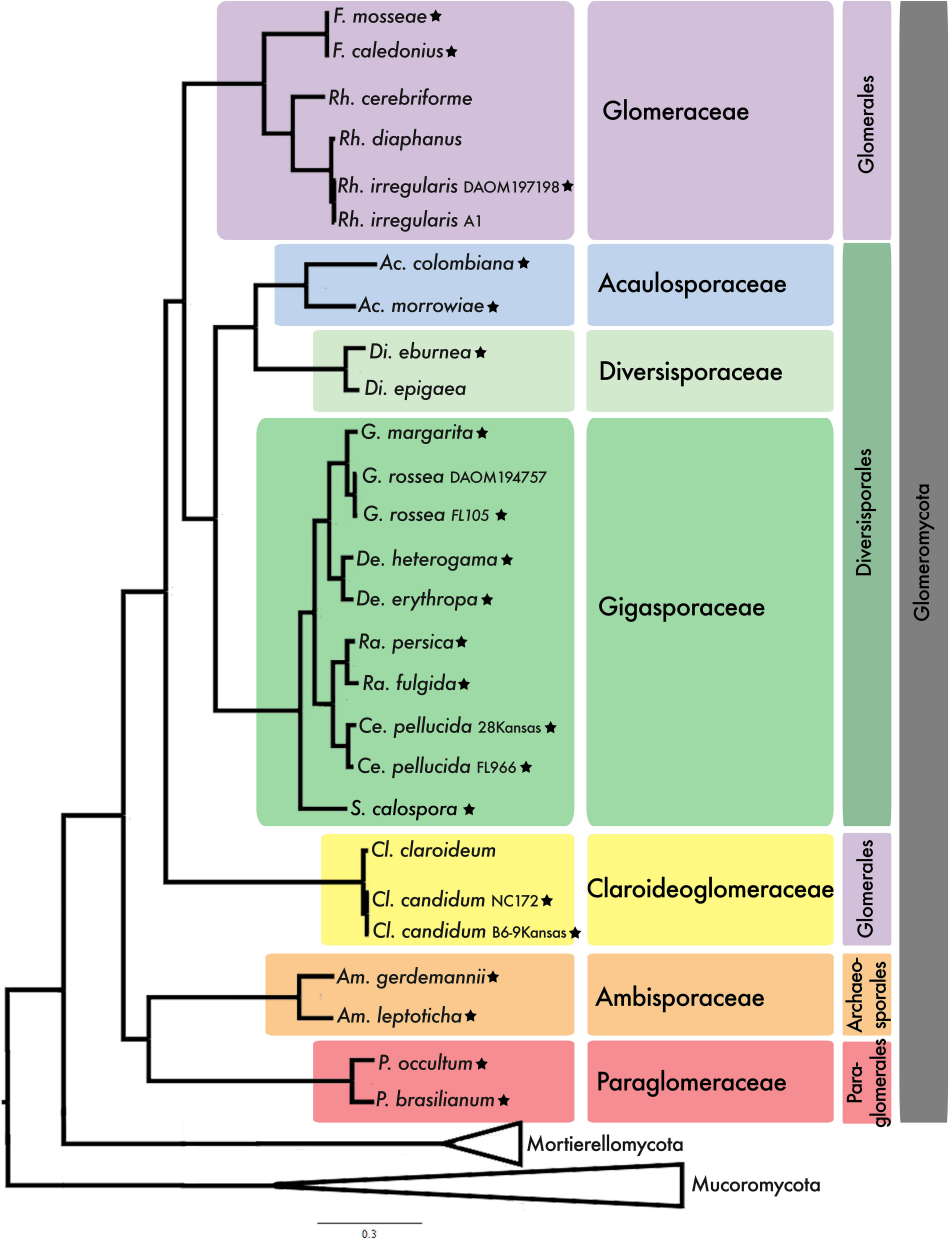
Best maximum likelihood tree inferred with RAxML from a concatenated alignment of 371 single copy orthologs shared among >50% of the taxa. The same topology was recovered using IQ-TREE and Bayesian inference. All nodes have a bootstrap support value of 100 in both analyses, and posterior probabilities of 1. Mucoromycota was used as outgroup. Stars following the taxon name mark newly sequenced strains from this study. Current taxonomic assignment based on Redecker et al. (2013) is color coded, at the levels of family and order. Strain identifers are included in the taxa label when more than one node has the same species name. See expanded tree in Supplementary Figure 7.

Two more datasets were designed to explore the relationships within Glomeromycota. One of them included published transcriptomic data from nine genera of AM fungi (Beaudet et al., 2018). However, only 17 SCOs shared among >50% of the taxa were retrieved (Supplementary Table 5). This low number is likely resulting from the fact that the transcriptomic dataset is less complete, ranging from 22 to 87% BUSCO completeness with an average of 57% across nine species (Beaudet et al., 2018), compared to the genomic data generated in our study which ranged from 44 to 95%, averaging at 83% across 21 strains included in the phylogenomic analysis. The phylogenetic placement of the strains with transcriptomic data is consistent with the placement of our newly sequenced strains (Supplementary Figure 6), but due to their low BUSCO values and little overlap of SCOs, the trancriptomic data was excluded from further analysis without decreasing the taxonomic breadth, while allowing us to work with a more comprehensive set of SCOs. The other dataset included published genome data and 21 newly sequenced strains of AM fungi, as well as representatives from Mortierellomycota and Mucoromycota. A concatenated alignment of 371 SCOs shared among >50% of the taxa produced a total alignment of 144,177 amino acids. All represented Glomeromycota families form well-supported monophyletic lineages in both ML and ASTRAL analyses (Figure 1; Supplementary Figure 7). This supports available phylogenetic inferences based on a combination of morphology and rDNA data (Redecker et al., 2013). We found, however, that the order Glomerales is polyphyletic, with Glomeraceae recovered as sister to the order Diversisporales (100% BS), while the family Claroideoglomeraceae forms a sister clade to the two (Figure 1; Supplementary Figure 7). The ASTRAL analysis recovered these same relationships but with low support (95% MLBS; 0.73 LPP) (Supplementary Figure 7). The ASTRAL analysis showed that the quartet gene tree frequencies of the three possible topologies were very similar (q1 = 0.37, q2 = 0.33, q3 = 0.30), and a polytomy could not be rejected with this dataset (*p*-value = 0.31) (Supplementary Figure 8).

### Exploring Conflicting Topologies

To further study contentious relationships within Glomeromycota (Claroideoglomeraceae, Glomeraceae, and Diversisporales), three datasets including only members of Glomeromycota, but with different taxon and/or gene sampling were produced (Supplementary Table 5). The first included 27 taxa with 1,737 SCOs present in >50% of the taxa (Supplementary Tables 2, 3). This dataset produced a concatenated alignment of 702,801 amino acids. The second included the same taxa, but only the 31 SCOs present in all taxa and produced a concatenated alignment of 15,443 amino acids. The third dataset included a selection of 15 *de novo* assembled Glomeromycota genomes with the highest quality (Supplementary Tables 3, 4) that represent all families with at least one species per genus. This last dataset was used to obtain a greater number of SCOs shared among all taxa. It included 799 SCOs, which resulted in a concatenated alignment of 476,329 amino acids.

**Figure 2:**
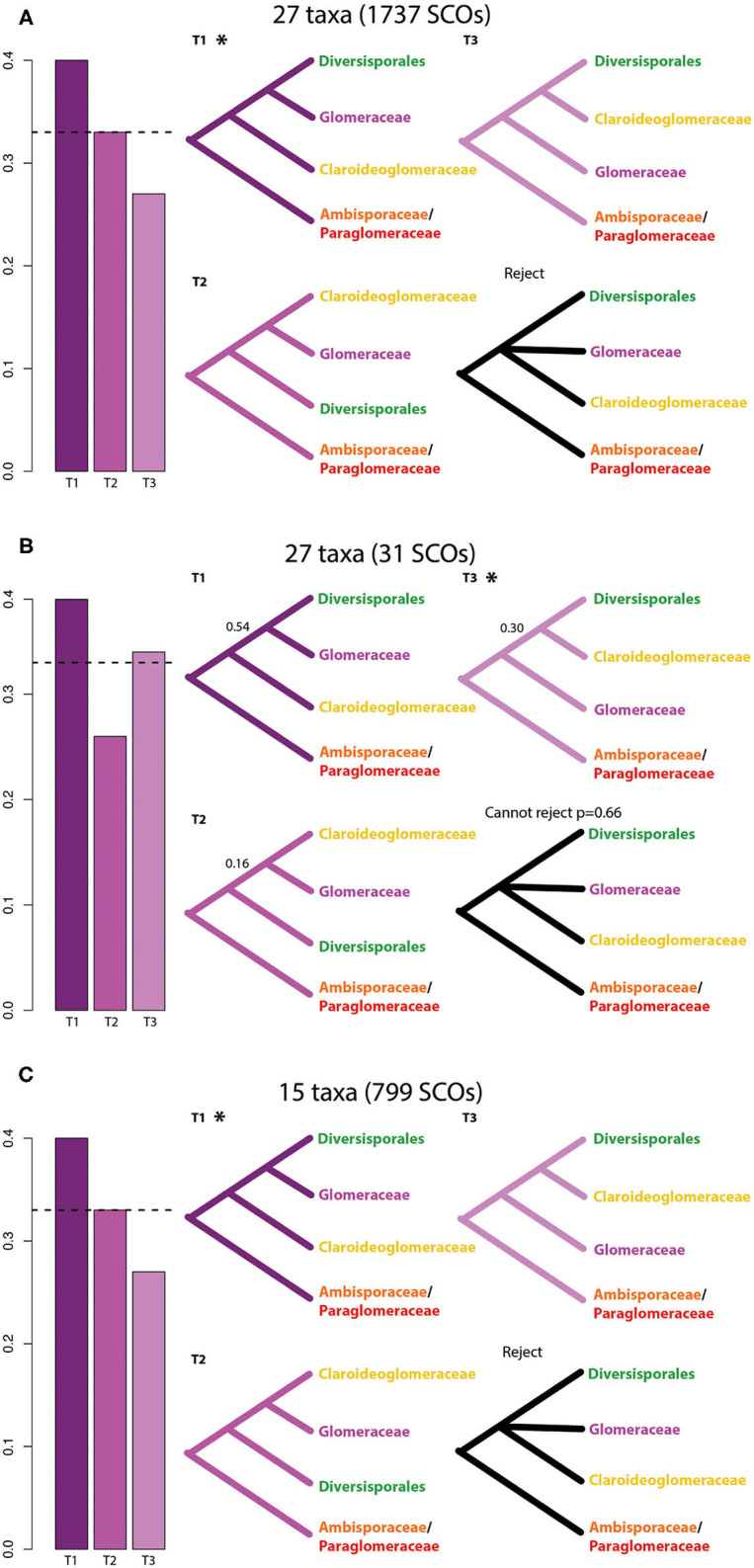
Evaluation of support among individual gene trees for alternative hypotheses of the relationships within Glomeromycota based on three datasets. **(A)** Glomeromycota dataset with single copy orthologs (SCOs) that are present in >50% of the taxa (27 taxa/1737 SCOs). **(B)** Glomeromycota dataset including SCOs that are present in all the taxa (27 taxa/31 SCOs). **(C)** Glomeromycota dataset with a selection of 15 taxa (see methods and Supplementary Table 4) including SCOs that are present in all taxa. Bar graphs represent the gene tree quartet frequencies for three possible branching orders within Glomeromycota. T1 corresponds to the ASTRAL topology, T2 and T3 correspond to alternative topologies in ASTRAL. Dashed horizontal lines marked the expectation of a hard polytomy. The topologies inferred with the concatenation-based method (Maximum Likelihood) are marked with an asterisk (^*^). Local posterior probabilities are indicated only when below 1.0.

Claroideoglomeraceae was well-supported as sister to Glomeraceae and Diversisporales in both the ML (100% BS) and ASTRAL (100% MLBS; 1.0 LPP) phylogenies, with the datasets that included 1,727 and 799 SCOs (Supplementary Figures 9, 10). The quartet-based analyses supported this branching (q1 = 0.4; q2 = 0.33; q3 = 0.27), for both datasets (Figures 2A,C), and a polytomy was rejected (p-value = 0). The same topology was weakly recovered in the ASTRAL analysis of the dataset with 31 SCOs (36% MLBS; 0.54 LPP) (Supplementary Figure 11), but the ML analysis inferred Claroideoglomeraceae as sister to Diversisporales with weak support (59% BS) (Supplementary Figure 11A). The quartet gene tree frequencies favored Glomeraceae as sister to Diversisporales (q1 = 0.39, q2 = 0.26, q3 = 0.34), but a polytomy could not be rejected with this dataset (*p*-value = 0.66) (Figure 2B). One of the alternative topologies that is recovered by rDNA genes (Supplementary Figures 1, 2), where Claroideoglomeraceae is sister to Glomeraceae in a monophyletic Glomerales, is frequently recovered but never statistically supported in any of the three datasets (Figure 2).

**Figure 3:**
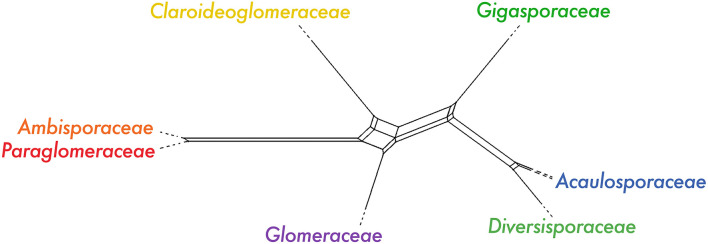
IQ-TREE network analysis visualized in SplitsTree5 with maximum dimension splits filter of 2, using the dataset containing all Glomeromycota taxa, and 1,737 SCOs shared among >50% of the taxa. See Supplementary Figure 13 for expanded network with full branch lengths.

In addition to the three possible topologies discussed above, there is a multitude of rare topologies among all 15 taxa, across the 799 single gene trees for SCOs shared among all taxa. These topologies are visualized using DensiTree (Bouckaert and Heled, 2014), in which the single gene trees are stacked on top of each other (Supplementary Figure 12). DensiTree shows that most genes support the topology, in which we recovered Glomeraceae as a sister group of Diversisporales, followed by the topology in which Glomerales is recovered as a monophyletic clade (Supplementary Figure 12), which is consistent with the quartet-based analysis (Figure 2). Across all analyses described above, we found consistent support for the polyphyly of Glomerales and a new hypothesis for the evolutionary relationships among families of Glomeromycota (Figures 1, 2). However, a phylogenomic network of the datasets with 1,737 and 799 SCOs revealed a clear reticulation at the base of the tree, indicating that the early evolutionary relationships cannot be resolved with the available data (Figure 3; Supplementary Figures 13, 14). Future studies may shed light on the processes behind these relationships.

## Discussion

Glomeromycota encompass all known AM fungi with their characteristic life cycle involving an obligate association with plants (Bonfante and Venice, 2020) as well as the exceptional fungal taxa *Geosiphon pyriformis* which forms a mutualistic symbiosis with the cyanobacteria *Nostoc punctiforme* (Malar et al., 2021). In the current study we present a four-fold increase in the number of AM fungal genomes available, which was achieved thanks to the development of a workflow for genome assembly from multiple individually amplified and sequenced nuclei (Montoliu-Nerin et al., 2020).

The current workflow for generating *de novo* reference genomes of AM fungi was developed by our team to circumvent the need for culturing AM fungi for genomic studies (Montoliu-Nerin et al., 2020). Read mapping of data from 24 individually amplified and sequenced *Rh. irregularis* DAOM197198 nuclei demonstrates near complete coverage of the published *Rh. irregularis* DAOM197198 v.2.0 reference genome (Supplementary Table 6), suggesting that separate amplification of multiple nuclei compensates for uneven amplification of individual nuclei. Consistent recovery of orthogroups in our *de novo* genome assembly of *Rh. irregularis* DAOM197198 (Supplementary Table 7) and evidence that mostly identical BUSCO genes are recovered from both assemblies provides further support that the presented workflow generates gene sequence data suitable for phylogenomic analysis. We anticipate that the release of these novel genome assemblies will become an important resource for the future study of AM fungi, supplementing already available AM fungal genomes (Tisserant et al., 2013; Lin et al., 2014; Chen et al., 2018; Kobayashi et al., 2018; Morin et al., 2019; Sun et al., 2019; Montoliu-Nerin et al., 2020).

Our phylogenomic analysis revealed a well-supported species tree for AM fungi. The relation of Glomeromycota to its two closest sister lineages, Mucoromycota and Mortierellomycota, had not yet been resolved with strong support, and based on previously available data the relation was best described as a polytomy (Li et al., 2021). Interestingly, with the addition of a considerable number of AM fungal genomes presented in this study we can still not reject a polytomy (Supplementary Figure 8). This further highlights the need for increased taxon sampling in the sister lineages of Glomeromycota, particularly in Mortierellomycota. Within Glomeromycota, we find that the seven family level lineages included in the analysis represent well-supported monophyletic lineages. Furthermore, while the order Diversisporales, including three families, was recovered as monophyletic we found that the order Glomerales with the two families Glomeraceae and Claroideoglomeraceae was not. Comprehensive phylogenetic studies with wide taxon sampling representing AM fungi have thus far mostly used rDNA sequences (Redecker et al., 2013) and recover Glomerales as monophyletic based on these markers. Similarly, our phylogenetic reconstructions using only the extracted rDNA operon from the *de novo* assembled genomes support Glomerales as monophyletic (Supplementary Figure 2). Glomerales was previously found to be polyphyletic in phylogenomic analyses using spore transcriptomic data from nine AM fungal species, where *Claroideoglomus* was recovered as sister to Ambispora and Paraglomus (Beaudet et al., 2018). In contrast to Beaudet et al. (2018), we recovered Claroideoglomeraceae as a sister to Glomeraceae and Diversisporales (Figures 1, 2). Previous phylogenomic studies using whole genomic data had not yet observed this topology due to limited taxon sampling (Morin et al., 2019).

The placement of Glomeraceae as a sister group of Diversisporales is well-supported in the phylogenies inferred using a concatenated dataset (Figure 1), as well as using a coalescence-based method (Supplementary Figure 7). Our analysis based on 27 Glomeromycota taxa and 1,737 SCOs that are present in >50% of the taxa strongly supported Claroideoglomeraceae as sister to Glomeraceae and Diversisporales. The dataset that included only 31 SCOs that are present in all taxa, showed low support for this relationship and a polytomy could not be rejected. These inconclusive results are most likely due to the small number of genes included in this dataset. It has been shown that phylogenetic inference can be robust to missing data (Wiens, 2003; Wiens and Morrill, 2011), therefore we expect that a more comprehensive set of SCOs, even when not present in all taxa, will provide a more accurate phylogenetic reconstruction than a complete dataset representing few genes. However, by analyzing a dataset with the 15 best assemblies, including a representative of each genus, we demonstrated that the use of assemblies with low completeness (based on BUSCO values) does not impact the phylogenetic inference.

It is possible that the topological discordances are due to incomplete lineage sorting (Maddison and Knowles, 2006), caused by long coalescence times which complicates the assessment of an accurate evolutionary history. Different topologies could also result from gene flow among AM fungal lineages. Documented gene family expansions correlated with genome size in AM fungi (Tang et al., 2016), could distort phylogenetic histories since gene expansions and contractions can cause misidentification of SCOs, resulting in alignments between paralogs present as single copy with different evolutionary origins and histories. A better understanding on how variation in gene content and copy number variation influenced the different topologies could be achieved with a deeper phylogenetic study into the whole repertoire of paralogs, moving one step further from SCOs, which would also allow us to look more closely into the possible correlation between gene function and different evolutionary histories.

## Conclusions

In the current study we present a considerable increase in the number of AM fungal genome assemblies available, thanks to the development of single nuclei sequencing and *de novo* assembling in AM fungi that we recently developed. As demonstrated for *Rh. irregularis*, variation in sequencing depth and coverage of single nuclei due to MDA, was accounted for in our *de novo* genome assemblies that provide a satisfactory representation of the genome content even when the assemblies generated are fragmented. Not all targeted species could be sorted, amplify or assemble equally well and species-specific method development may be required for a more complete dataset. Nevertheless, we present a phylogenomic analysis of AM fungi based on the most comprehensive taxon sampling across Glomeromycota to date. Our results support current family-level classification and concur in one strongly supported topology. In this topology, the order Glomerales is polyphyletic, with the family Glomeraceae being recovered as a sister group to the order Diversisporales, with Claroideoglomeraceae as their sister group. The new genome data presented cover seven families of the phylum Glomeromycota and are expected to be a valuable contribution to the AM fungal research community.

## Data Availability Statement

The datasets presented in this study can be found in online repositories. The names of the repository/repositories and accession number(s) can be found below: https://www.ebi.ac.uk/ena, PRJEB45340 and linked public OSF repository doi: 10.17605/OSF.IO/2ZYP4.

## Author Contributions

MM-N initiated the project together with AR and JB and developed the analysis together with MS-G and HJ. CB did the nuclei sorting and whole genome amplification. MM-N and MS-G performed the bioinformatic analyses. VK developed the annotation workflow which was ran by MM-N and MS-G. MM-N and MS-G wrote the manuscript with AR and HJ with input from all the authors. All authors contributed to the article and approved the submitted version.

## Funding

Funding for this project was provided by ERC (678792). VK was financially supported by the Knut and Alice Wallenberg Foundation as part of the National Bioinformatics Infrastructure Sweden at SciLifeLab. Open access funding provided by Uppsala University and funding for maintenance of AMF cultures by NSF 2027458.

## Conflict of Interest

The authors declare that the research was conducted in the absence of any commercial or financial relationships that could be construed as a potential conflict of interest.

## Publisher's Note

All claims expressed in this article are solely those of the authors and do not necessarily represent those of their affiliated organizations, or those of the publisher, the editors and the reviewers. Any product that may be evaluated in this article, or claim that may be made by its manufacturer, is not guaranteed or endorsed by the publisher.
