# Supplementary material for: In-depth Phylogenomic Analysis of Arbuscular Mycorrhizal Fungi Based on a Comprehensive Set of de novo Genome Assemblies
Source: Front Fungal Biol. 2021 Sep 29;2:716385. doi: 10.3389/ffunb.2021.716385 (PMC10512289; doi:10.3389/ffunb.2021.716385)
Supplement: Supplementary file 1 [file Data_Sheet_1.PDF]

Supplementary material

Supplementary tables

Table S1. Table of all isolates attempted for sequencing.
Table S2. Published data included in phylogenomic analysis.
Table S3. Whole genome assembly information for all *de novo* sequenced isolates.
Table S4. Complete BUSCO statistics for all *de novo* sequenced isolates
Table S5. Overview of six datasets with different taxon and gene sampling
Table S6. Assembly coverage of reads mapped to two assemblies of *Rh. irregularis* DAOM197198
Table S7. Summary statistics from OrthoFinder across two assemblies of *Rh. irregularis* DAOM197198

Supplementary figures

Figure S1. SSU rDNA phylogeny to confirm isolate identity.
Figure S2. Phylogenetic analysis of complete rDNA operon of 21 isolates this study.
Figure S3. Visualization of best ML tree including Glomeromycota and its sister lineages with Dikarya as outgroup.
Figure S4. ML tree A) Expanded version of in Fig. S3 B) IQ-TREE and C) ASTRAL.
Figure S5. Quartet gene tree frequencies for relationships among the three sister phyla.
Figure S6. Best ML tree including transcriptomic data from Beaudet *et al.*, 2018.
Figure S7. 371 SCOs. A) expanded version of ML tree in Fig. 1, B) ASTRAL.
Figure S8. Quartet gene tree frequencies for relationships within Glomeromycota.
Figure S9. Glomeromycota 1,737 SCOs. A) best ML tree B) ASTRAL.
Figure S10. 15 selected taxa in Glomeromycota. A) best ML tree B) ASTRAL.
Figure S11. 31 SCOs in Glomeromycota. A) best ML tree B) ASTRAL.
Figure S12. DensiTree based on IQ-TREE individual gene trees.
Figure S13. Network from IQ-TREE, using 1,737 single gene trees.
Figure S14. Network from IQ-TREE, using 799 single gene trees.

Table S1. AM fungal taxa for which *de novo* genome sequencing was attempted, organized according to the classification on the INVAM homepage (2020-08-01). The whole genome amplification (WGA) kit used for amplification of the single nuclei is indicated for each isolate. Number (Nr.) of spores indicates how many spores were pooled to extract nuclei for FACS, and number of nuclei shows how many nuclei samples were individually selected for sequencing, resulting in the same number of libraries successfully sequenced.

Family	Genus	Species	Strain	Collection[3]	Nuclei sorted[4]	WGA	Nr. spores	Nr. nuclei
**Glomeraceae**	Funneliformis	*Funneliformis mosseae*	87-6 pot B 2015	Kansas		Epicentre	~15	20
		Funneliformis caledonius	UK204	INVAM		Qiagen	~15	24
	Septoglomus	*Septoglomus viscosum*	MD215	INVAM		Qiagen	~15	20
		Septoglomus constrictum	KS890	INVAM		Qiagen	~15	-
	Glomus	*Glomus microaggregatum*	UT126B	INVAM		Qiagen	~15	-
		Glomus gold	KS906B	INVAM		Qiagen	~15	-
	Rhizophagus	*Rhizophagus irregularis*	DAOM197198	Canada		Qiagen	1	24
		Rhizophagus intraradices	FL208A	INVAM		Epicentre	1	24
**Acaulosporaceae**	Acaulospora	*Entrophospora infrequence* [1]	110 2015	Kansas	No	na	~15	-

		Entrophospora infrequence [1]	CA203	INVAM		Qiagen	~15	-
		Acaulospora colombiana	CL356	INVAM		Qiagen	~15	15
		Acaulospora morrowiae	CL551	INVAM		Qiagen	~15	19
**Diversisporaceae**	Diversispora	*Diversispora epigaea*	AZ150B	INVAM		Qiagen	~15	7
		Diversispora eburnea	AZ414A	INVAM		Qiagen	~15	24
**Gigasporaceae**	Gigaspora	*Gigaspora margarita*	120-4 pot B 10/14	Kansas		Qiagen	~15	24
		Gigaspora rosea	FL105	INVAM		Epicentre	~15	20
	Dentiscutata	*Dentiscutata heterogama*	IL203A	INVAM		Qiagen	~15	7
		Dentiscutata erythropus	MA453B	INVAM		Qiagen	~15	24
	Cetraspora	*Cetraspora pellucida*	FL966	INVAM		Qiagen	~15	24
		Cetraspora pellucida [2]	28 12/20/2015	Kansas		Epicentre	~15	17
	Racocetra	*Racocetra persica*	MA461A	INVAM		Qiagen	~15	24

		Racocetra fulgida	IN212	INVAM		Qiagen	~15	22
	Scutellospora	*Scutellospora calospora*	AU212A	INVAM		Epicentre	~15	13
**Claroideoglomeraceae**	Claroideoglomus	*Claroideoglomus candidum*	NC172	INVAM		Epicentre	1	24
		Claroideoglomus candidum	(CCK) pot B 6-9	Kansas		Qiagen	7	24
**Paraglomeraceae**	Paraglomus	*Paraglomus occultum*	IA702	INVAM		Epicentre	~15	23
		Paraglomus brasilianum	BR232B	INVAM		Epicentre	~15	20
**Archaeosporaceae**	Archaeospora	*Archaeospora trappei*	IL203B	INVAM	No	na	~15	-
		Archaeospora schencki	CL383	INVAM		Qiagen	~15	24
**Ambisporaceae**	Ambispora	*Ambispora leptoticha*	FL130A	INVAM		Qiagen	~15	22
		Ambispora gerdemannii	MT106	INVAM		Qiagen	~15	24

1. Taxonomic placement of *Entrophspora infrequences* remains unresolved
2. The strain was previously named *Scutellospora pellucida*. It is the same culture as INVAM collection *Cetraspora pellucida* (INVAM IN211)
3. Collections from where the strains were obtained: Kansas: James D. Bever's lab, University of Kansas, USA; INVAM: International culture collection of (vesicular) arbuscular mycorrhizal fungi, West Virginia University, Morgantown, WV, USA; Canada: Agriculture and Agri-food Canada.
4. Shadowed boxes indicate successful nuclei sorting.

Table S2. Published data used in phylogenomic analyses. Annotations of the whole genome assemblies were downloaded from their original source. Final column shows in which figures each isolate was included.

Phyla	Species	Isolate	Publication	Used for Fig.
Glomeromycota	*Rhizophagus irregularis*	A1	(Chen *et al.*, 2018)	1-2, S3-S9, S13
	Rhizophagus diaphanus	MUCL43196	(Morin *et al.*, 2019)	1-2, S3-S9, S13
	Rhizophagus cerebriforme	DAOM227022	(Morin *et al.*, 2019)	1-2, S3-S9, S13
	Rhizophagus irregularis	DAOM234181	(Beaudet *et al.*, 2018)	S6
	Funneliformis mosseae	DAOM236685	(Beaudet *et al.*, 2018)	S6
	Acaulospora morrowiae	CR315B	(Beaudet *et al.*, 2018)	S6
	Diversispora epigaea	IT104	(Sun *et al.*, 2019)	1-2, S3-S9, S13
	Diversispora versiforme	W475-40	(Beaudet *et al.*, 2018)	S6
	Gigaspora rosea	DAOM194757	(Morin *et al.*, 2019)	1-2, S3-S9, S13
	Racocetra castanea	BEG 1	(Beaudet *et al.*, 2018)	S6
	Scutellospora calospora	IL209	(Beaudet *et al.*, 2018)	S6
	Claroideoglomus claroideum	DAOM234280	(Beaudet *et al.*, 2018)	S6
	Claroideoglomus claroideum	SA101	(Montoliu-Nerin *et al.*, 2020)	1-2, S3-S14
	Ambispora lepototicha	JA116	(Beaudet *et al.*, 2018)	S6
	Paraglomus brasilianum	DAOM240472	(Beaudet *et al.*, 2018)	S6
Mortierellomycota	*Mortierella elongata*	AG 77	(Uehling *et al.*, 2017)	1, S3-S6
	Lobosporangium transversale	NRR 3116	(Mondo *et al.*, 2017a)	1, S3-S6
Mucoromycota	*Endogone sp.*	FLAS 59071	(Chang *et al.*, 2019)	1, S3-S6

	Jimgerdemannia lactiflua	OSC 166217	(Chang *et al.*, 2019)	1, S3-S6
	Jimgerdemannia flammicorona	AD 002	(Chang *et al.*, 2019)	1, S3-S6
	Syncephalastrum racemosum	NRRL 2496	(Mondo *et al.*, 2017a)	1, S3-S6
	Lichtheimia corymbifera	JMRC FSU 9682	(Schwartze *et al.*, 2014)	1, S3-S6
	Hesseltinella vesiculosa	NRRL 3301	(Mondo *et al.*, 2017a)	1, S3-S6
	Absidia repens	NRRL 1336	(Mondo *et al.*, 2017a)	1, S3-S6
	Saksenaea vasiformis	B4078	(Chibucos *et al.*, 2016)	1, S3-S6
	Phycomyces blakesleeanus	NRRL 1555	(Corrochano *et al.*, 2016)	1, S3-S6
	Rhizopus microsporus var. microsporus	ATCC 52813	(Mondo *et al.*, 2017b)	1, S3-S6
	Rhizopus microsporus var. chinensis	CCTCCM 201021	(Wang *et al.*, 2013)	1, S3-S6
	Mucor circinelloides	CBS 277 49	(Corrochano *et al.*, 2016)	1, S3-S6
	Rhizopus delemar	RA99 880	(Ma *et al.*, 2009)	1, S3-S6
Basidiomycota	*Laccaria bicolor*	S238N-H82	(Martin *et al.*, 2008)	S3-S5
	Ustilago maydis	521, DSMZ14603	(Kämper *et al.*, 2006)	S3-S5
	Puccinia striiformis f. sp. tritici	104 E137 A-	(Schwessinger *et al.*, 2018)	S3-S5
Ascomycota	*Tuber melanosporum*	Mel28	(Martin *et al.*, 2010)	S3-S5
	Schizosaccharomyces pombe	-	(Wood *et al.*, 2002)	S3-S5
	Yarrowia lypolitica	FKP355	(Pomraning *et al.*, 2018)	S3-S5

Table S3. Whole genome assembly statistics for all *de novo* genome assemblies generated in this study. Showing results from the QUAST analysis (Estimated size, Size, #Contigs, N50, Largest contig and GC), estimated completeness analysis (BUSCO), results from gene prediction and repeat annotation pipeline (# Genes and (content), Repeat content). Final column list figures where taxon is included.
* Indicate that the strain removed due to unclear identity prior to phylogenomic analysis and only included in Fig. S1.

Species	Estimated size (Mb)	Size (Mb)	# Contigs	N50	Largest contig (Kb)	GC (%)	BUSCO (%)	# Genes and (content, Mb)	Repeat content (Mb)	Used for Fig.
F. mosseae	156.54	145.72	27,134	11,988	97.04	25.22	C: 86, F: 6	16,857 (42.65)	77.14	1-2, S1-S14
F. caledonius	150.38	146.16	31,052	9,933	81.73	26.00	C: 89, F: 4	17,946 (41.00)	75.48	1-2, S1-S14
Se. viscosum *	86.48	69.04	13,413	14,169	159.44	36.25	C: 90, F: 4	14,815 (37.81)	17.27	S1
Rh. irregularis DAOM197198	133.86	116.45	15,939	21,163	222.15	27.28	C: 94, F: 1	23,258 (68.13)	33.80	1-2, S1-S14
Rh. intraradices *	133.86	116.45	15,939	21,163	222.15	27.28	C: 94, F: 1	23,258 (68.13)	33.80	S1
Ac. colombiana	247.44	299.81	7,5131	8,208	86.29	29.39	C: 78, F: 9	14,505 (23.78)	240.52	1-2, S1-S14
Ac. morrowiae	154.35	218.77	64,585	6,189	54.03	27.89	C: 74, F: 14	18,394 (31.29)	159.05	1-2, S1-S9, S11, S13
Di. eburnea	87.85	65.11	9,803	22,612	184.86	25.84	C: 92, F: 3	12,017 (37.55)	25.62	1-2, S1-S14
Di. epigaea *	54.32	64.27	16,527	8,159	138.06	38.11	C: 76, F: 12	15,972 (29.58)	10.12	S1
G. margarita	492.60	574.57	173,647	5,875	76.23	27.40	C: 88, F: 3	46,492 (82.99)	378.98	1-2, S1-S14
G. rosea	200.66	240.31	90,143	4,479	50.41	28.74	C: 46, F: 13	26,343 (36.22)	154.42	1-2, S1-S9, S11, S13
De. heterogama	167.37	181.57	61,528	4,991	40.56	27.81	C: 44, F: 11	16,277 (23.62)	121.82	1-2, S1-S9, S11, S13
De. erythropus	248.76	293.54	73,964	7,545	61.80	28.34	C: 87, F: 4	28,764 (58.16)	190.17	1-2, S1-S14
Ra. persica	176.70	353.07	181,541	2,886	30.66	27.09	C: 47, F: 24	37,045 (36.01)	238.55	1-2, S1-S9, S11, S13
Ra. fulgida	212.27	295.99	105,307	4,839	74.97	28.40	C: 68, F: 14	19,906 (29.68)	205.34	1-2, S1-S14
Ce. pellucida 28 K[1]	214.21	226.81	79,972	4,932	51.05	26.61	C: 43, F: 12	18,058 (26.55)	164.40	1-2, S1-S9, S11, S13
Ce. pellucida FL966	418.72	435.08	90,930	10,017	77.05	25.97	C: 89, F: 5	22,053 (51.29)	325.42	1-2, S1-S14

S. callospora	143.92	147.04	50,548	4,999	47.38	26.36	C: 29, F: 15	11,479 (16.87)	107.94	1-2, S1-S9, S11, S13
Cl. candidum NC172	86.47	68.12	12,232	15,216	101.84	27.83	C: 88, F: 3	15,761 (42.66)	21.83	1-2, S1-S14
Cl. candidum B6-9K	87.88	69.90	12,603	15,877	114.29	27.86	C: 87, F: 4	16,088 (43.88)	22.68	1-2, S1-S14
Ar. schenckii *	86.41	89.56	17,380	12,048	123.55	36.28	C: 86, F: 8	19,726 (47.95)	24.04	S1
Am. gerdemannii	102.08	87.97	19,363	10,266	96.67	28.22	C: 90, F: 3	13,690 (33.64)	42.05	1-2, S1-S14
Am. leptoticha	163.75	197.20	58,506	5,886	99.48	23.62	C: 90, F: 3	14,642 (30.94)	144.29	1-2, S1-S14
P. occultum	49.53	50.06	8,053	16,033	146.03	36.55	C: 76, F: 7	11,385 (30.35)	10.57	1-2, S1-S9, S11, S13
P. brasilianum	61.76	58.47	7,115	21,894	153.20	36.59	C: 90, F: 3	11,842 (36.52)	15.09	1-2, S1-S14

1. The strain was named *Scutellospora pellucida* in Bever lab collection (K for Kansas). It is the same culture as INVAM collection *Cetraspora pellucida* (INVAM IN211)

Table S4. BUSCO statistics for all *de novo* genome assemblies generated using single nuclei sequencing and assembly of combined reads. Number of genes out of a total of 290 conserved single copy genes. Column "15 selected" marks the 15 genome assemblies selected for in-depth analysis of Glomeromycota.

Species	Complete single copy	Complete duplicate d	Fragmented	Missing	15 selected
F. mosseae	248	1	18	23	*
F. caledonius	256	2	12	20	*
Se. viscosum [3]	257	5	11	17	
Rh. irregularis	271	1	4	14	*
Rh. intraradices [3]	249	2	14	25	
Ac. colombiana	188	37	25	40	*
Ac. morrowiae	207	8	42	33	
Di. eburnea	265	3	9	13	*
Di. epigaea [3]	181	41	36	32	
G. margarita	252	4	9	25	*
G. rosea	131	1	38	120	
De. heterogama	127	1	31	131	
De. erythropus	246	5	13	26	*
Ra. persica	135	0	70	85	
Ra. fulgida	195	3	40	52	*
Ce. pellucida 28 K[1]	124	1	34	131	
Ce. pellucida FL966	255	4	14	17	*
S. calospora	84	1	42	163	
Cl. claroideum	224	31	13	22	*
Cl. candidum	222	33	10	25	*
Cl. candidum K[2]	223	30	12	25	*
Ar. schenckii [3]	200	50	23	17	
Am. gerdemannii	256	6	9	19	*
Am. leptoticha	254	5	10	21	*
P. occultum	219	3	40	52	
P. brasilianum	258	4	10	18	*

1. The strain was named *Scutellospora pellucida* in Bever lab collection (K for Kansas). It is the same culture as INVAM collection *Cetraspora pellucida* (INVAM IN211)
2. K is added to indicate that this is the *Claroideoglomus candidum* from Kansas (CCK) pot B 6-9 is from the Bever lab collection.
3. Phylogenetic placement based on rDNA SSU genes did not support the names assigned to these strains and they were thus removed from further analysis.

Table S5. Six datasets with different taxon and SCOs sampling were used for the phylogenomic analyses. For each dataset figure numbers are listed for results from the five types of phylogenomic analysis performed: maximum likelihood (ML), Bayesian, coalescent-based approach (ASTRAL), split networks in IQ_tree (Network) and topology visualization (DensiTree). Supplementary figures are indicated with S before the number.

**Dataset**	Number of taxa	% of taxa with shared SCOs	Number of SCOs	ML tree	Bayesian tree	Astral tree	Net-work	Densi-Tree
Dikarya + Glomeromycota + Mucoromycota + Mortierellomycota	47	50%	178	S3, S4A		S4B, S5		
Glomeromycota + transcriptome data + sister phyla	53	50%	17	S6				
Glomeromycota + sister phyla	41	50%	371	Fig 1, S7A	S7A	S7B, S8		
Glomeromycota	27	50%	1,737	S9A		Fig. 2A, S9B	S13	
Glomeromycota	27	100%	31	S11A		Fig. 2B, S11B		
Glomeromycota selected	15	100%	799	S10A		Fig. 2C S19B	S14	S12

Table S6. Mapping of single nuclei reads of *Rh. irregularis* DAOM197198 to the reference genome assembly v.2.0 (ref v.2.0) (Chen *et al.*, 2018), and the *de novo* assembly produced in this study (*de novo*). Percentage of mapped reads from each nucleus (1-24) and percentage of the assembly covered by the reads from 24 individually amplified and sequenced nuclei. With average in bold. Last two rows indicate % reads mapped and % of assembly covered when all reads are pooled before mapping, separately for 1x and 50x.

	% mapped reads		% of assembly covered (>= 1X)	
	ref v.2.0 (%)	*de novo* (%)	ref v.2.0 (%)	*de novo* (%)
Rh. irregularis - Nucleus 1	99.11	99.61	50.84	53.42
Rh. irregularis - Nucleus 2	99.35	99.58	25.83	26.72
Rh. irregularis - Nucleus 3	99.39	99.63	51.75	54.34
Rh. irregularis - Nucleus 4	99.27	99.62	54.31	57.16
Rh. irregularis - Nucleus 5	99.36	99.67	50.34	52.85
Rh. irregularis - Nucleus 6	99.06	99.52	52.90	55.68
Rh. irregularis - Nucleus 7	99.26	99.67	51.65	54.13
Rh. irregularis - Nucleus 8	93.52	99.65	39.68	41.49
Rh. irregularis - Nucleus 9	99.31	99.62	64.93	68.37
Rh. irregularis - Nucleus 10	99.39	99.69	65.17	68.58
Rh. irregularis - Nucleus 11	99.34	99.65	66.51	70.01
Rh. irregularis - Nucleus 12	99.45	99.68	20.97	22.20
Rh. irregularis - Nucleus 13	99.30	99.67	62.98	66.20
Rh. irregularis - Nucleus 14	92.37	92.79	53.95	56.48
Rh. irregularis - Nucleus 15	99.14	99.53	85.38	90.25
Rh. irregularis - Nucleus 16	98.83	99.71	47.19	49.65
Rh. irregularis - Nucleus 17	98.80	99.58	47.36	49.62
Rh. irregularis - Nucleus 18	99.44	99.63	41.46	43.41
Rh. irregularis - Nucleus 19	99.33	99.65	64.42	67.94
Rh. irregularis - Nucleus 20	99.24	99.57	48.54	50.98
Rh. irregularis - Nucleus 21	99.45	99.65	37.99	39.70
Rh. irregularis - Nucleus 22	99.36	99.68	44.36	46.40
Rh. irregularis - Nucleus 23	99.24	99.60	32.81	34.04
Rh. irregularis - Nucleus 24	99.07	99.42	39.91	41.85
**Average across 24 nuclei**	**98.72**	**99.34**	**50.05**	**52.56**
24 nuclei combined 1X	98.67	99.24	94.79	100
24 nuclei combined 50X	98.67	99.24	91.49	97.21

Table S7. OrthoFinder statistics for orthogroups shared between *Rh. irregularis* DAOM197198 reference genome v.2.0 (Chen *et al.*, 2018) and the *de novo* assembly of the same strain produced in this study (Both) followed by numbers for each assembly.

Number of "species" (i.e. genome assemblies)	Both	v.2.0	*de novo*
Number of genes	49,451	26,183	23,268
Number of genes in orthogroups	43,894	23,429	20,465
Number of unassigned genes	5,557	2,754	2,803
Percentage of genes in orthogroups	88.8	89.5	88.0
Percentage of unassigned genes	11.2	10.5	12.0
Number of orthogroups	13,908	13,528	13,505
Number of species-specific orthogroups	783	403	380
Number of genes in species-specific orthogroups	4,264	2,638	1,626
Percentage of genes in species-specific orthogroups	8.6	10.1	7.0
Mean orthogroup size	3.2		
Median orthogroup size	2.0		
G50 (assigned genes)	3		
G50 (all genes)	2		
O50 (assigned genes)	3,092		
O50 (all genes)	4,324		
Number of orthogroups with all species present	13,125		
Number of single-copy orthogroups	10,138		

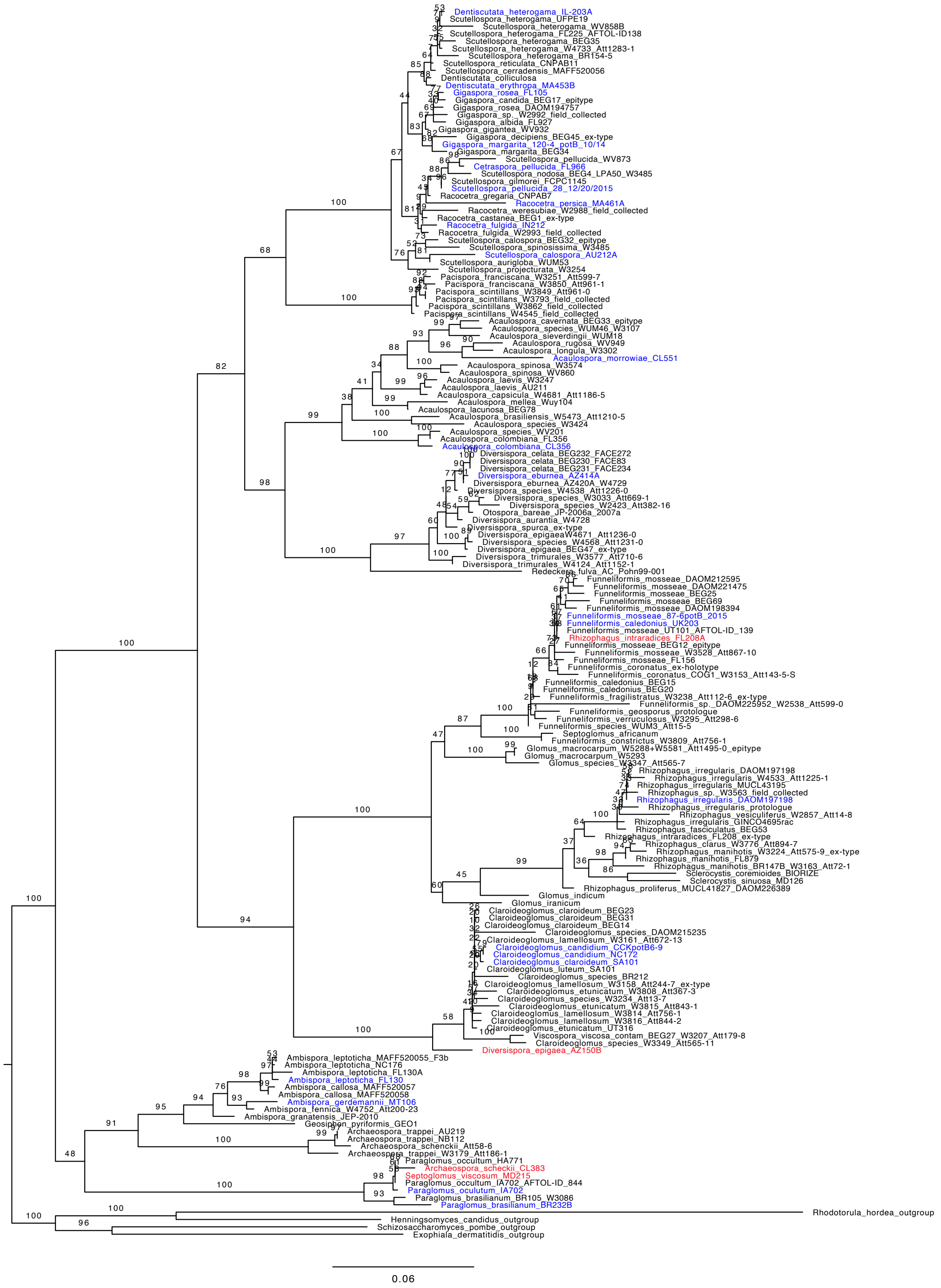

Figure S1. Best maximum likelihood RAxML tree based on a ribosomal small subunit (SSU) alignment from Krüger *et al.,* (2012) including the SSU sequences from the newly generated assemblies (blue and red). Isolates sequenced in the current study are labeled according to initial information from culture collections (Supplementary Table 1). Taxon names in red indicate that the isolate was excluded from further analysis because of suspected contamination or misidentification due to inconsistent placement based on taxon name. Taxa in blue were included in the downstream analyses. Branch labels show bootstrap support (100 replicates).

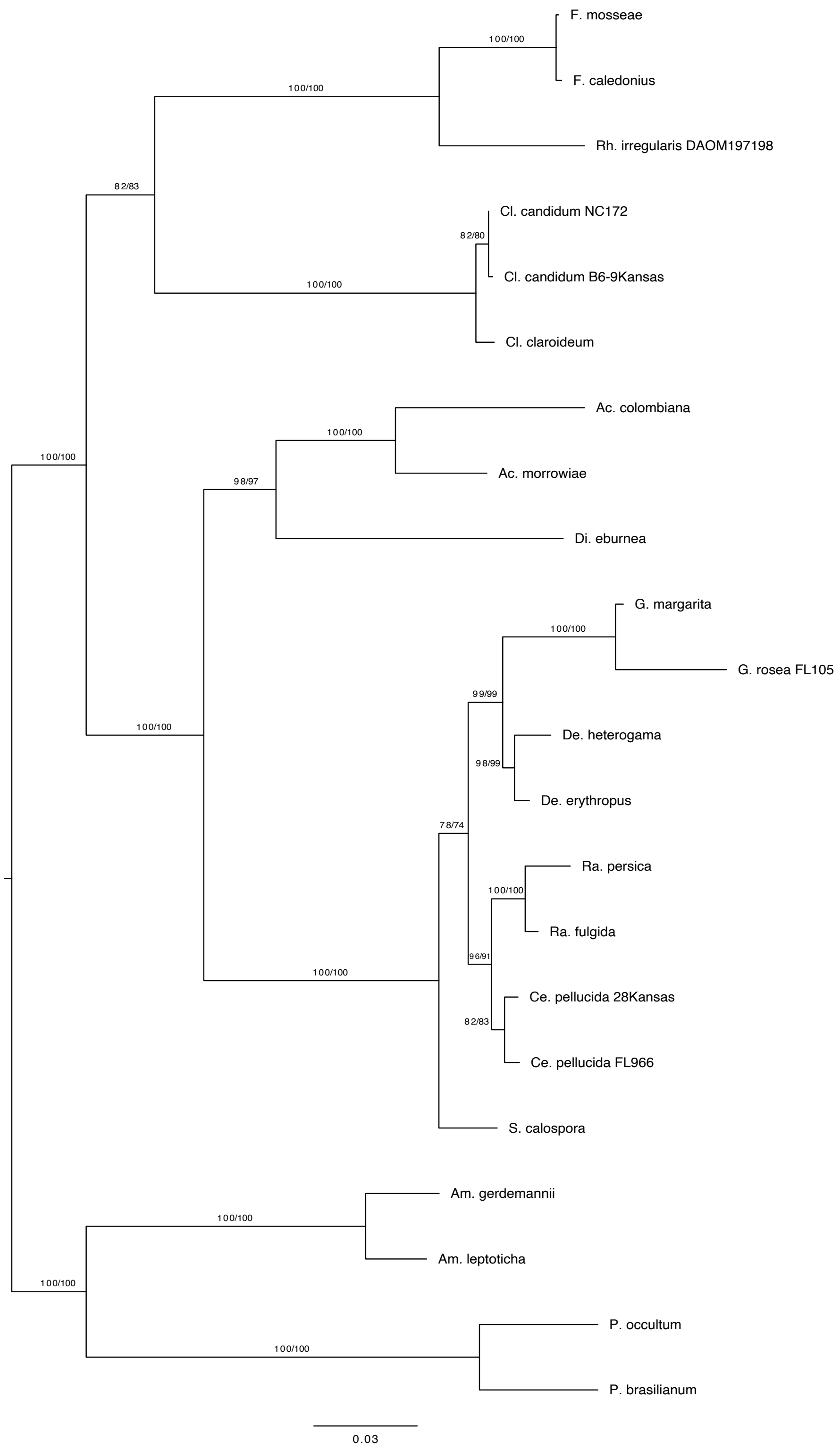

Figure S2. Best maximum likelihood RAxML tree from an alignment of the rDNA sequences (SSU+5.8s+LSU) of the newly assembled genomes. The same topology was obtained from an IQ-TREE analysis. Bootstrap values (1000 replicates) are shown above or next to the branches (RAxML/IQ-TREE). Full species names are presented in Table S1. Strain identifiers are indicated when two or more strains of the same species are included. (Supplementary Table 1, 2).

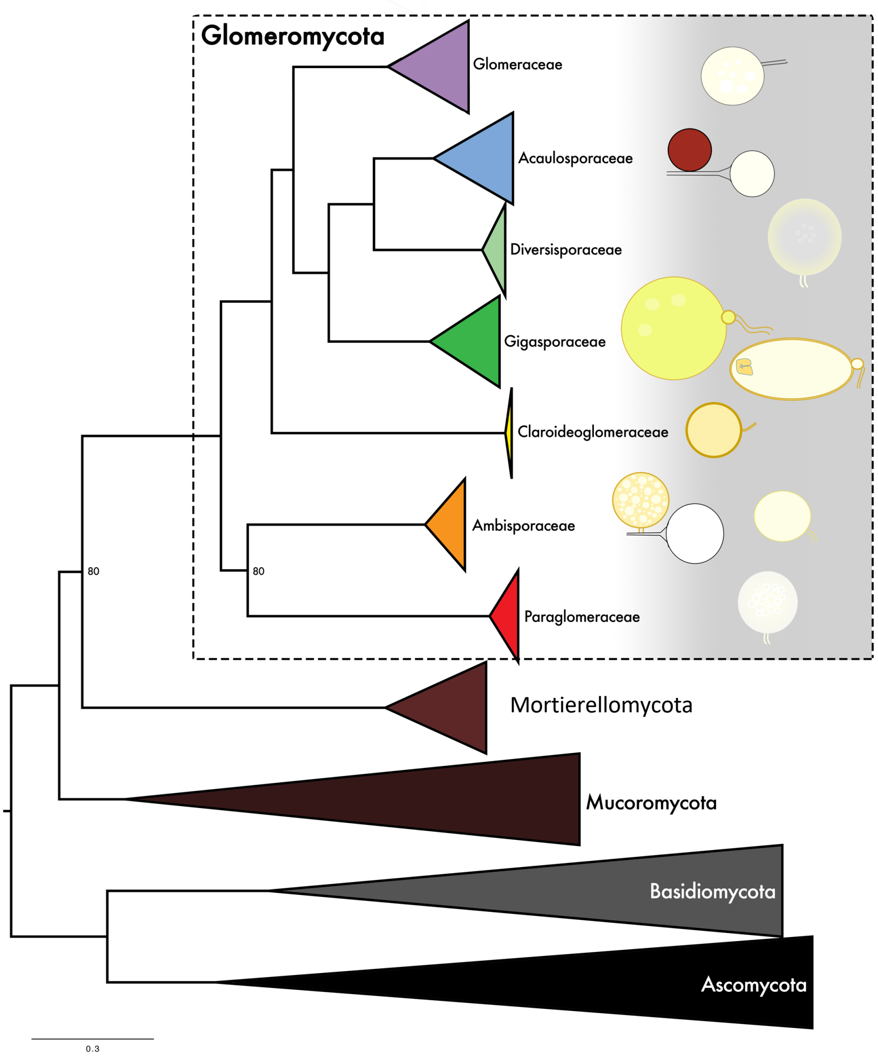

Figure S3. Best maximum likelihood RAxML tree from a concatenated alignment of 178 single copy orthologs shared among > 50% of the taxa. All branches have bootstrap support of 100 unless indicated. Basidiomycota and Ascomycota (Dikarya) were used as outgroup. All phyla are collapsed with the exception of Glomeromycota, where seven families are visualized in the dashed box. Typical spore morphologies are schematically illustrated to the right of each family and not drawn to scale. For expanded tree see Supplementary Figure 4A.

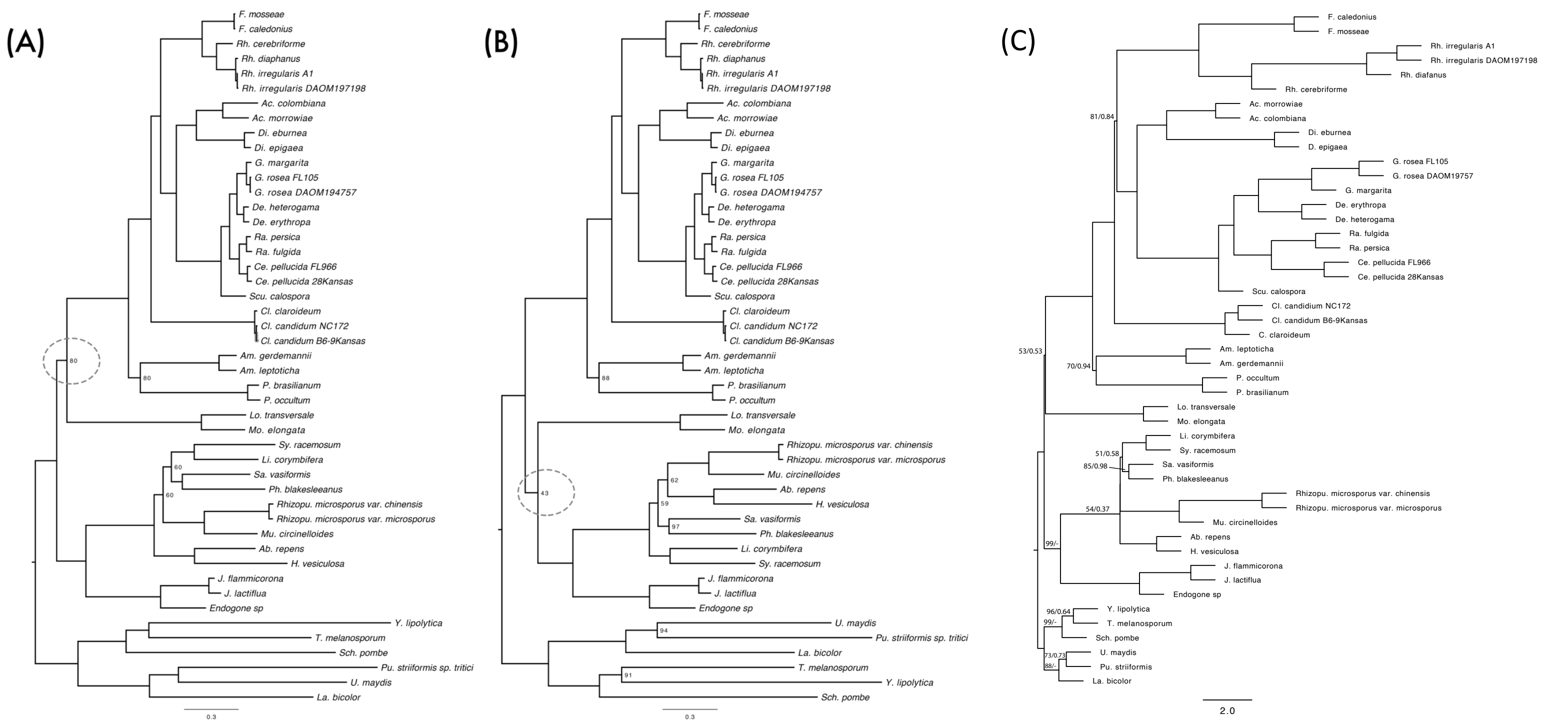

Figure S4. Phylogeny of Glomeromycota, Mucoromycota and Mortierellomycota including members of Dikarya as outgroup. Best maximum likelihood **(A)** RAxML and **(B)** IQ-TREE tree from a concatenated alignment of 178 single copy orthologs shared among >50% of the taxa. All branches have bootstrap support of 100 unless indicated in the tree. Circled nodes indicate conflict between the two maximum likelihood topologies, in which Mortierellomycota is recovered as a sister group of Glomeromycota (in A) or of Mucoromycota (in B). **(C)** ASTRAL phylogeny based on 178 individual gene trees inferred with IQ-TREE. Multi-locus bootstrapping and local posterior probabilities are indicated at the nodes when below 100 or 1.0 (MLBS/LPP). Full species names are presented in Supplementary Table 1, 2. Strain identifiers are indicated when two or more strains of the same species are included.

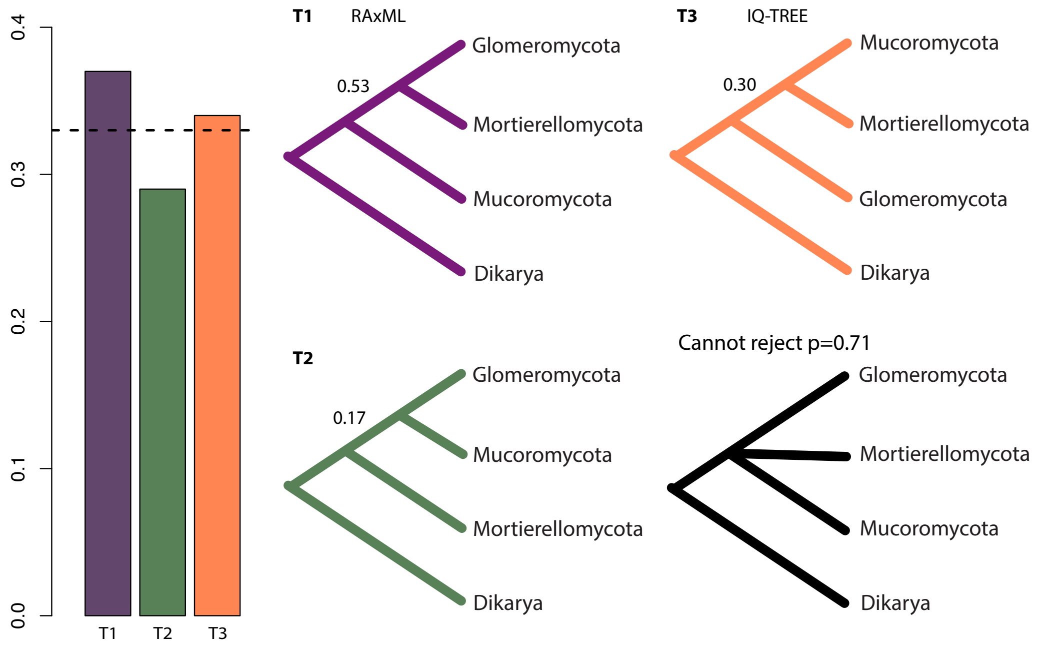

Figure S5. Evaluation of support among individual gene trees for alternative hypotheses of the relationships among Glomeromycota, Mucoromycota and Mortierellomycota. Bar graphs represent the gene tree quartet frequencies for three possible branching orders. T1 corresponds to the ASTRAL topology, T2 and T3 correspond to alternative topologies in ASTRAL. The topologies recovered by RAxML and IQ-TREE are indicated. Dashed horizontal lines marked the expectation of a hard polytomy. Local posterior probabilities are indicated only when below 1.0.

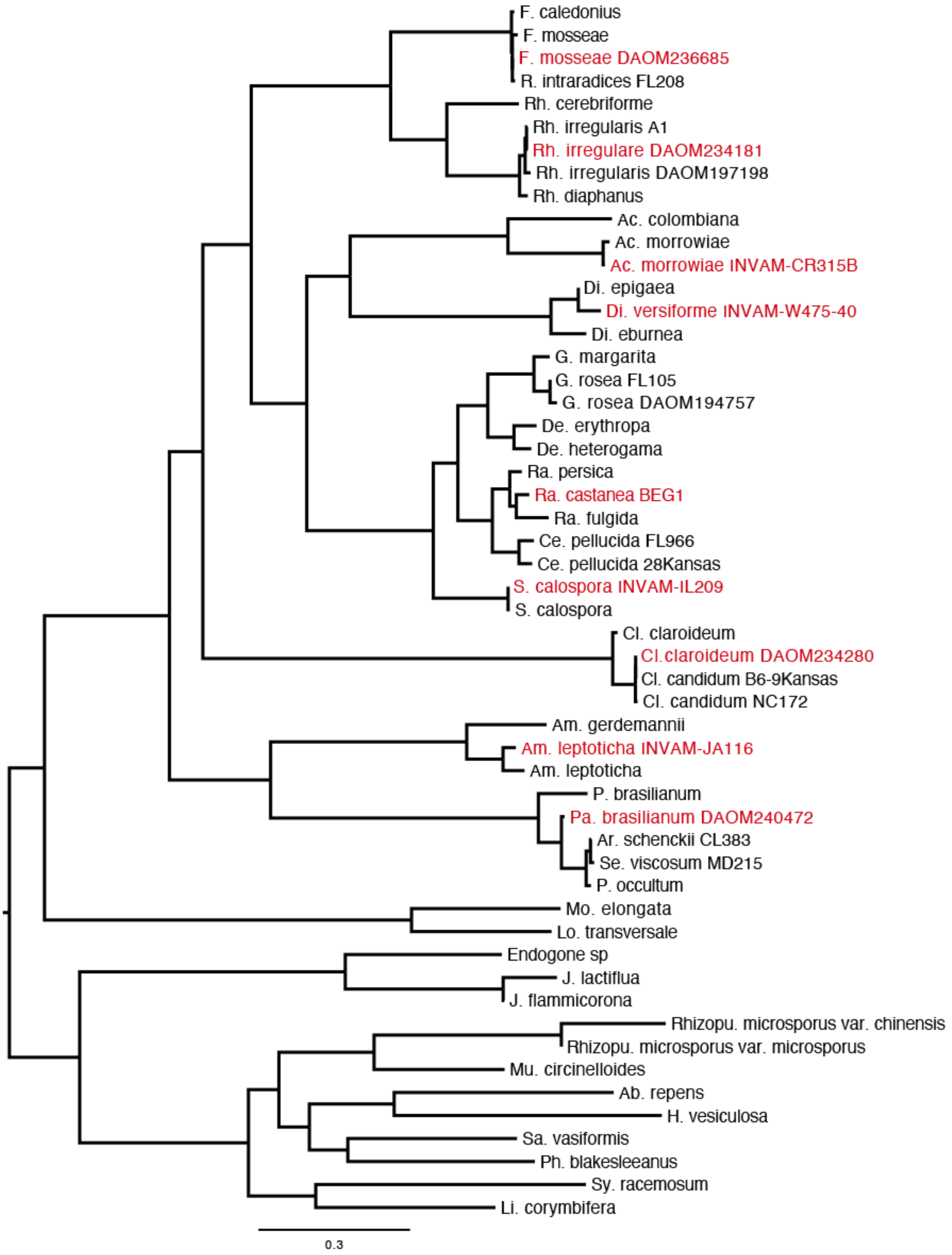

Figure S6. Best maximum likelihood RAxML tree from a concatenated alignment of 17 single copy orthologs shared among >50% of the taxa. All branches have bootstrap support of 100. Taxa in red show transcriptomic data from Beaudet *et al.*, 2018 and include species name and strain identifier. The transcriptomic data from Beaudet *et al.*, 2018 was not included in downstream analysis. Strain identifiers are indicated when two or more strains of the same species are included (Supplementary Table 1, 2).

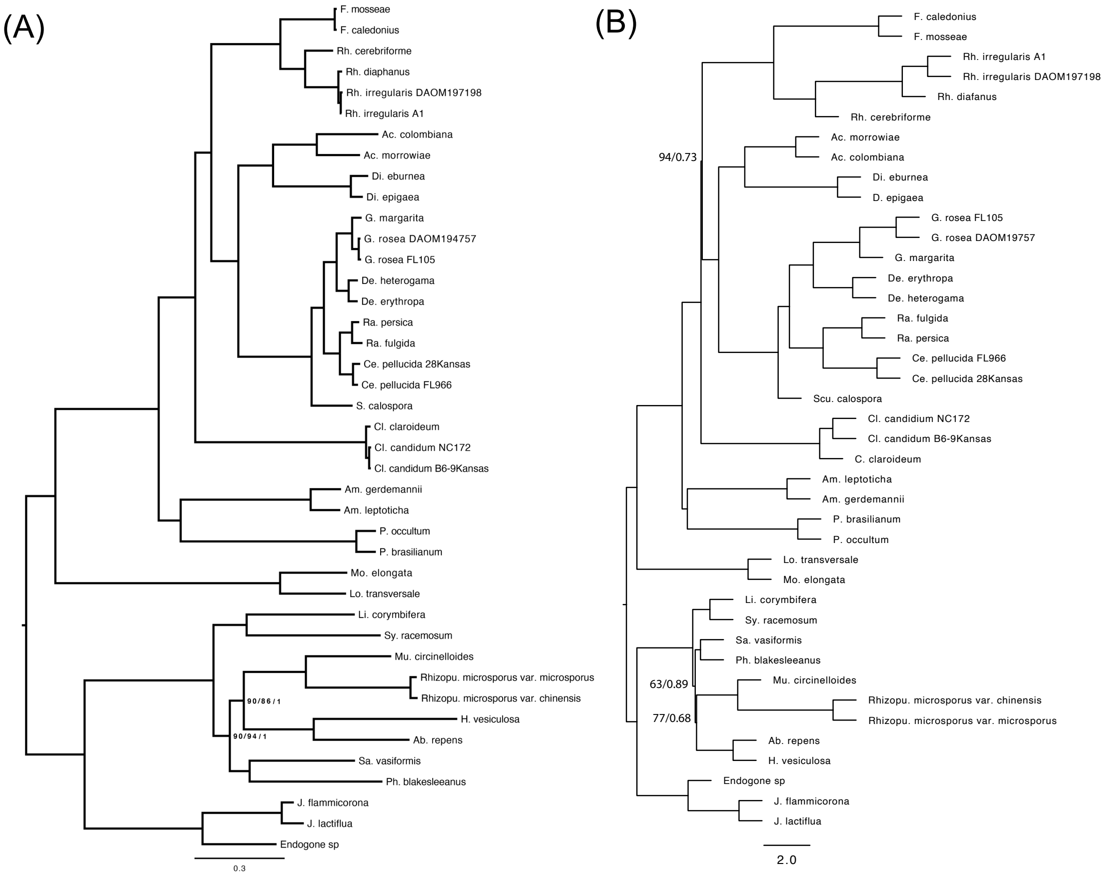

Figure S7. Phylogeny of Glomeromycota including Mortierellomycota and Mucoromycota as outgroup **(A)** Expanded tree corresponding to Figure 1. Best maximum likelihood tree inferred with RAxML from a concatenated alignment of 371 single copy orthologs shared by >50% of the taxa. The same topology was recovered using IQ-TREE and Bayesian inference. Support values are indicated at the nodes when below 100 or 1.0 (RAxML/IQ-TREE/Bayesian). **(B)** ASTRAL phylogeny based on 371 individual gene trees inferred with IQ-TREE. Support values are indicated at the nodes when below 100 or 1.0 (MLBS/LPP). Strain identifiers are included when two or more strains of the same species are included (Supplementary Table 1, 2).

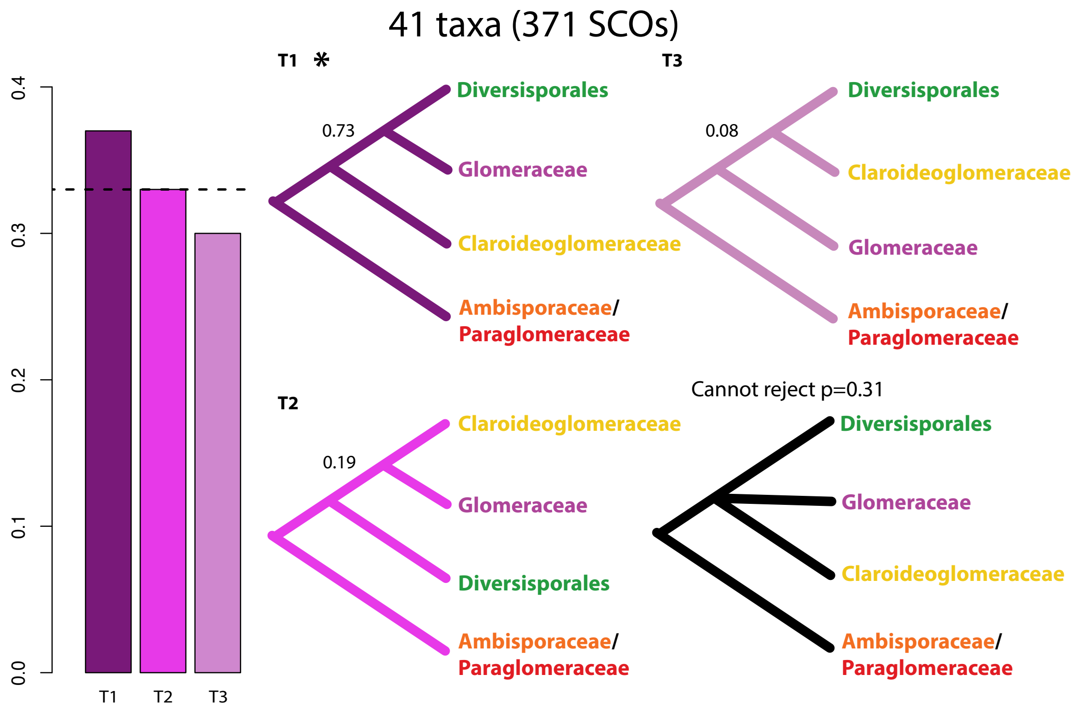

Figure S8. Evaluation of support among individual gene trees for alternative hypotheses of the relationships within Glomeromycota based on the dataset of Glomeromycota plus its sister phyla. Bar graphs represent the gene tree quartet frequencies for three possible branching orders. T1 corresponds to the ASTRAL topology, T2 and T3 correspond to alternative topologies in ASTRAL. Dashed horizontal lines marked the expectation of a hard polytomy. Local posterior probabilities are indicated only when below 1.0.

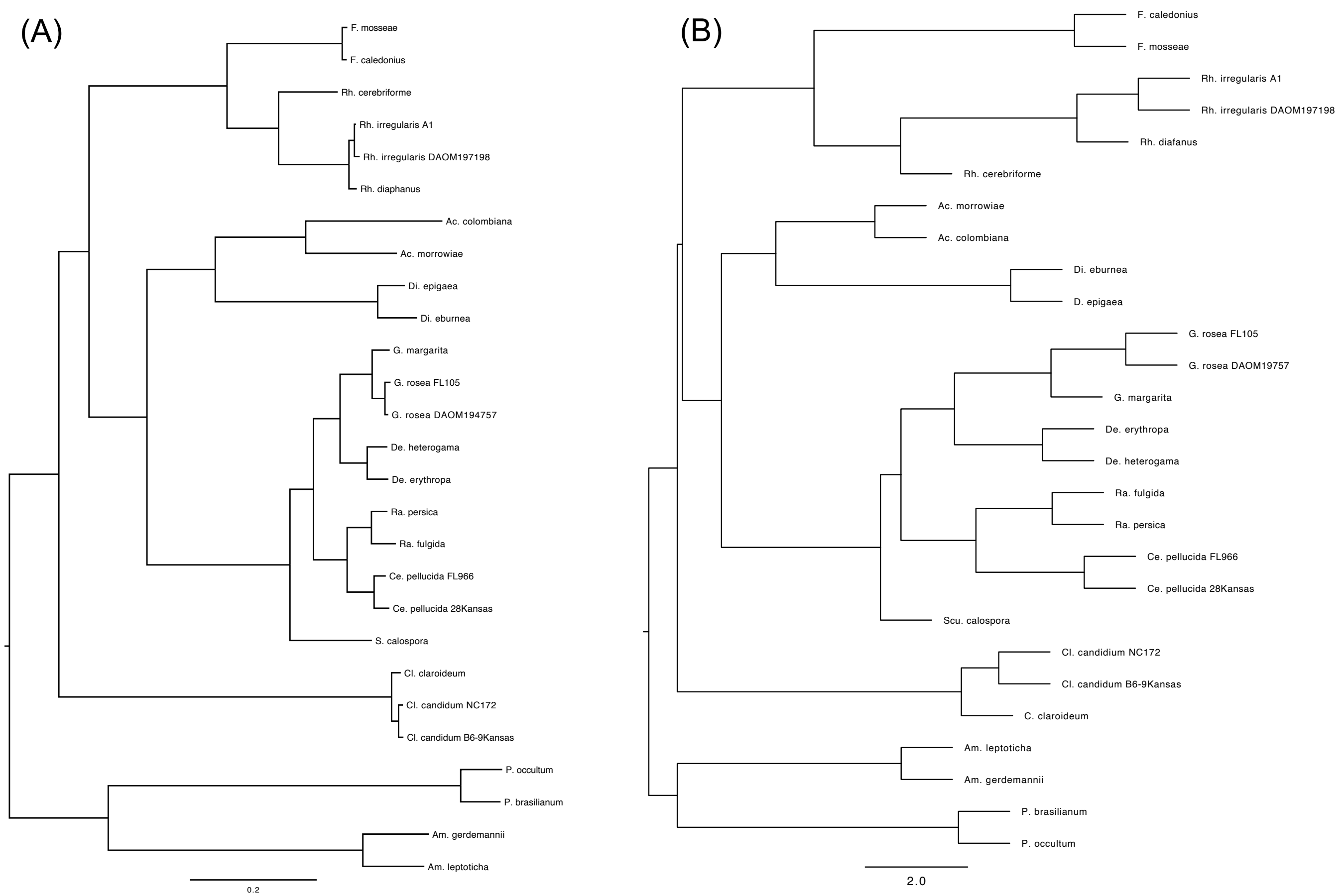

Figure S9. Phylogeny of Glomeromycota based on 1,737 single copy orthologs shared among 27 taxa. **(A)** Best maximum likelihood tree inferred with RAxML from a concatenated alignment of 1,737 single copy orthologs shared among >50% of the taxa. The same topology was recovered using IQ-TREE. All nodes have bootstrap value support of 100. **(B)** ASTRAL phylogeny based on 1,737 individual gene trees inferred with IQ-TREE. All nodes have multi-locus bootstrap support of 100 and local posterior probabilities of 1.0. Strain identifiers are included when two or more strains of the same species are included (Supplementary Table 1).

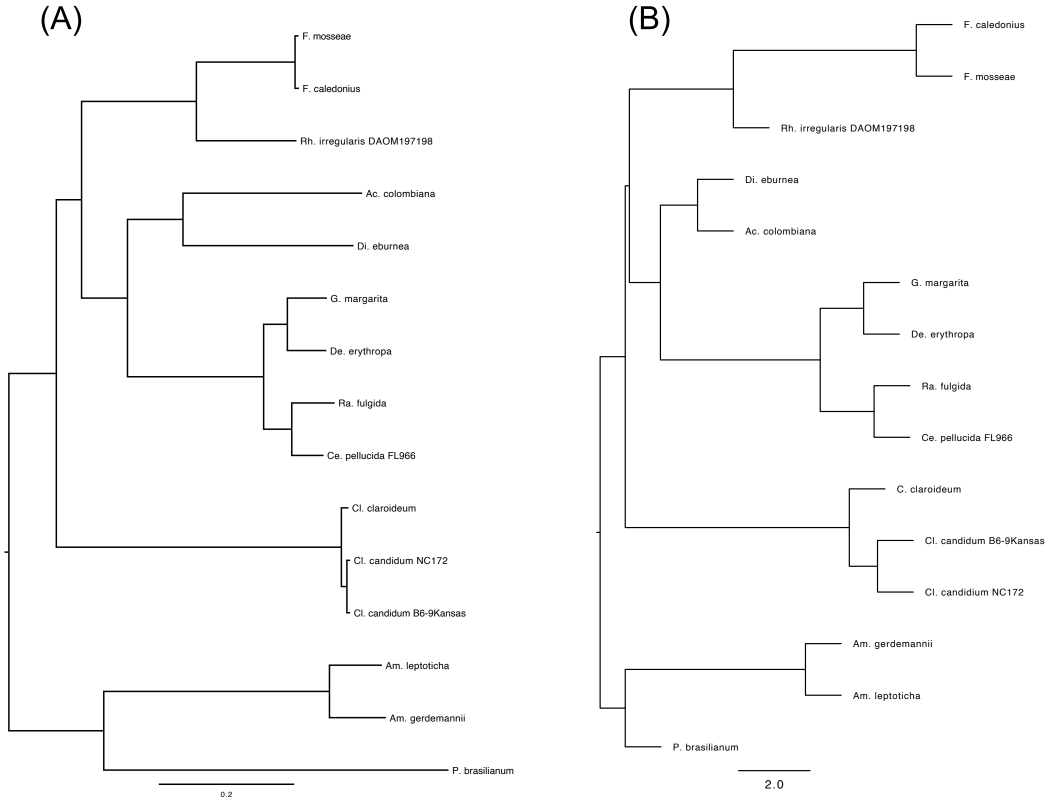

Figure S10. Phylogeny of Glomeromycota based on 799 single copy orthologs shared among 15 selected taxa (Supplementary Table 4). **(A)** Best maximum likelihood tree inferred with RAxML from a concatenated alignment of 799 single copy genes shared among all taxa. Same topology was recovered using IQ-TREE. All nodes have bootstrap value support of 100. **(B)** ASTRAL phylogeny based on 799 individual gene trees inferred with IQ-TREE. All nodes have multi-locus bootstrap support of 100 and local posterior probabilities of 1.0. Strain identifiers are included when two or more strains of the same species are included (Supplementary Table 1).

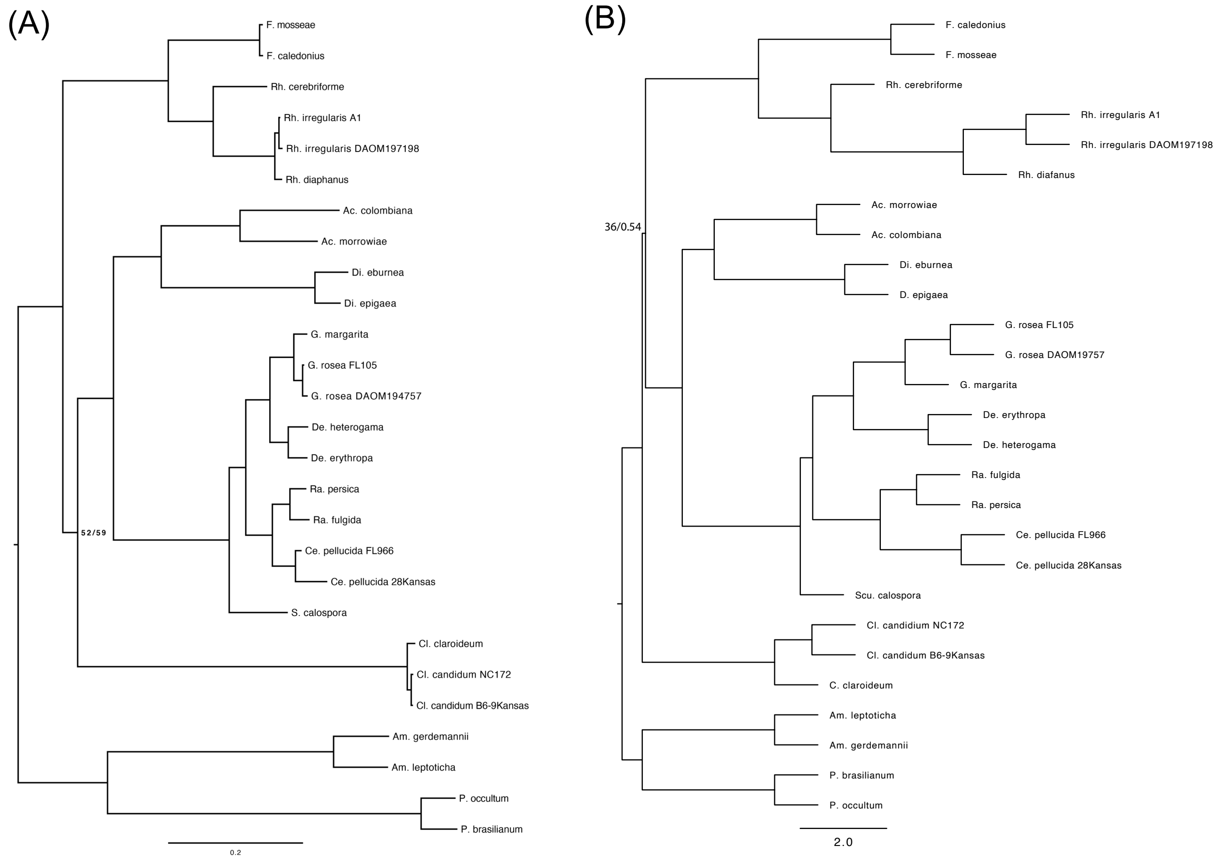

Figure S11. Phylogeny of Glomeromycota based on 31 single copy orthologs shared all 27 taxa. **(A)** Best maximum likelihood tree inferred with RAxML from a concatenated alignment of 31 single copy genes shared among all taxa. Same topology was recovered using IQ-TREE. Support values are indicated at the nodes when below 100 (RAxML/IQ-TREE). **(B)** ASTRAL phylogeny based on 31 individual gene trees inferred with IQ-TREE. Support values are indicated at the nodes when below 100 or 1.0 (MLBS/LPP). Strain identifiers are included when two or more strains of the same species are included (Supplementary Table 1).

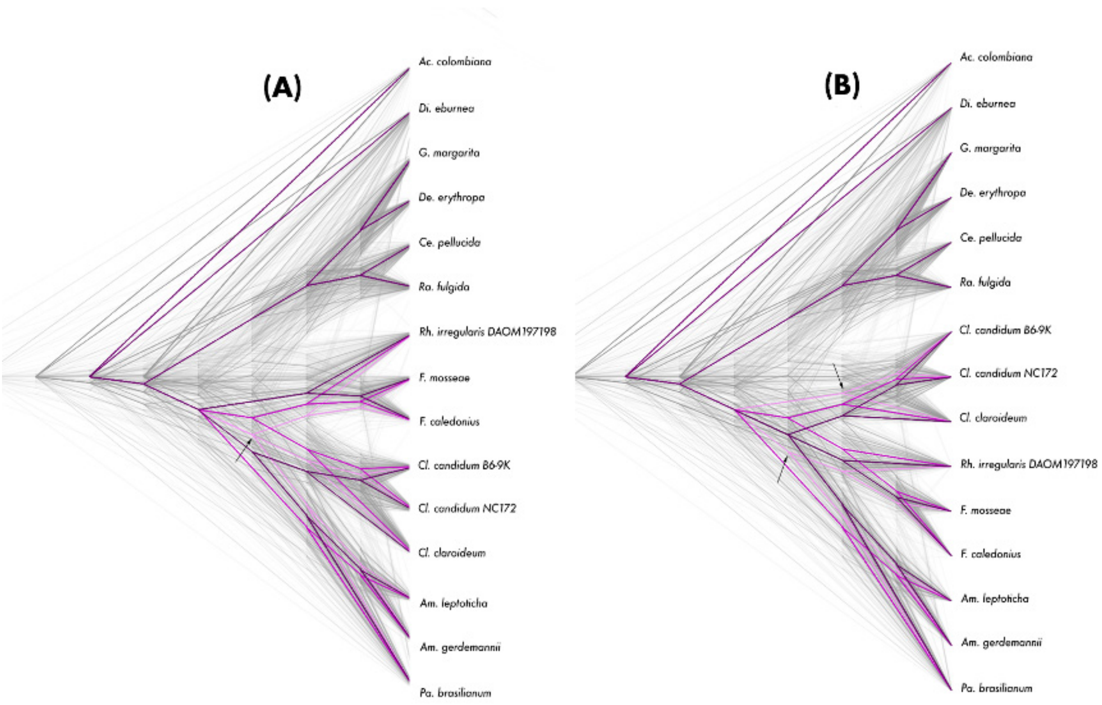

Figure S12. DensiTree based on 799 individual gene trees inferred with IQ-TREE for 15 selected taxa with black arrows indicating topology 3. The order of taxa is rearranged from (A) to (B) to better visualize topology 3. Full species names are presented in Supplementary Table 1 and strain identifiers are included when two or more strains of the same species are included.

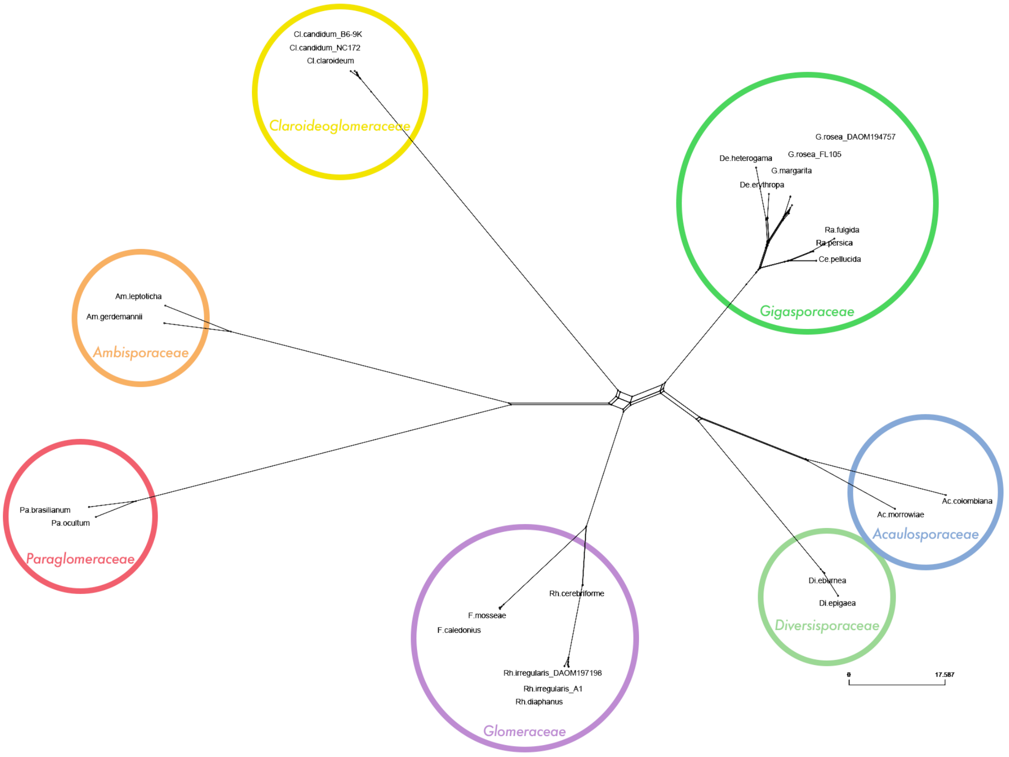

Figure S13. Split network produced with IQ-TREE network analysis and visualized in SplitsTree5 with maximum dimension splits filter of 2, using the dataset containing 27 Glomeromycota taxa, and 1,737 SCOs shared among >50% of the taxa. Branch lengths between families (Circled and color coded according to Figure 1 and Supplementary Table 1) are visualize compared to Figure 3 that provides a zoom in of the reticulation at the base. Strain identifiers are included when two or more strains of the same species are included (Supplementary Table 1).

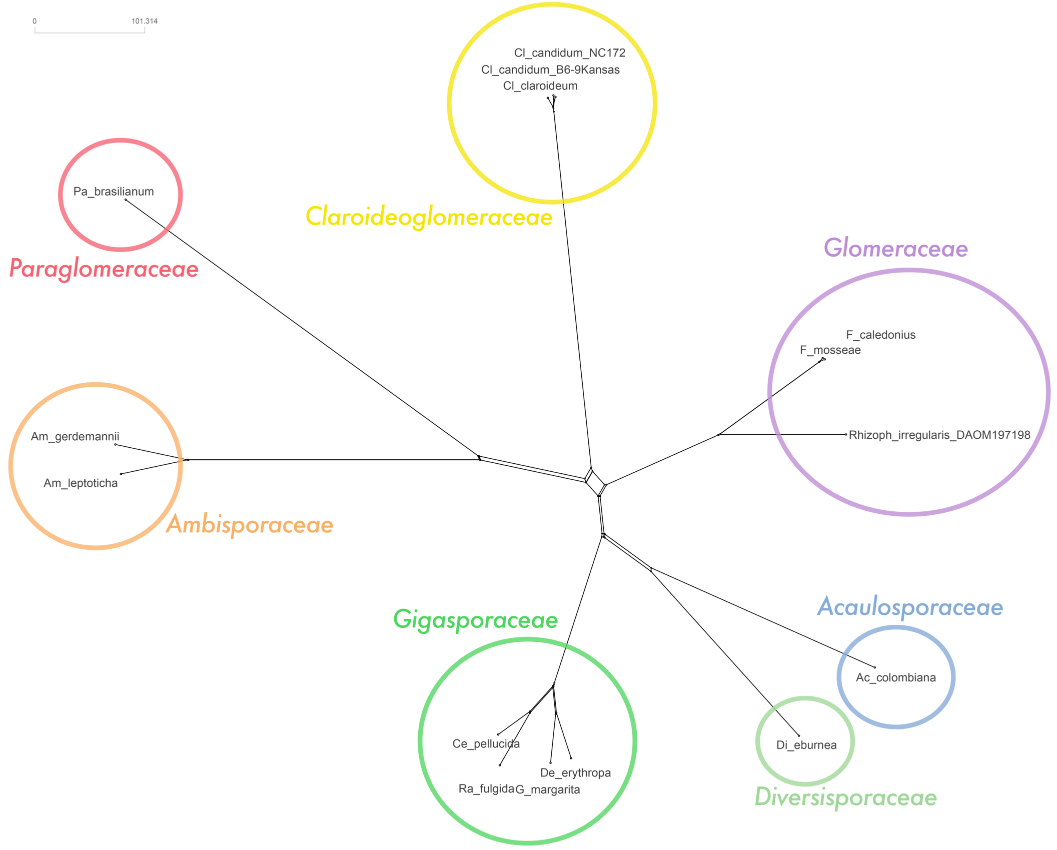

Figure S14. Network from IQ-TREE network analysis, using 799 single gene trees shared among 15 selected taxa (Supplementary Table 4), visualized in SplitsTree5, with a maximum dimension filter of 2. Families are circled and color coded according to Figure 1 and Supplementary Table 1 where full species names are presented.

References

Beaudet D, Chen EC, Mathieu S, Yildirir G, Ndikumana S, Dalpé Y, Séguin S, Farinelli L, Stajich JE, Corradi N. 2018. Ultra-low input transcriptomics reveal the spore functional content and phylogenetic affiliations of poorly studied arbuscular mycorrhizal fungi. *DNA Research* **25**(2): 217-227.

Chang Y, Desirò A, Na H, Sandor L, Lipzen A, Clum A, Barry K, Grigoriev IV, Martin FM, Stajich JE. 2019. Phylogenomics of Endogonaceae and evolution of mycorrhizas within Mucoromycota. *New Phytologist* **222**(1): 511-525.

Chen EC, Morin E, Beaudet D, Noel J, Yildirir G, Ndikumana S, Charron P, St-Onge C, Giorgi J, Krüger M. 2018. High intraspecific genome diversity in the model arbuscular mycorrhizal symbiont *Rhizophagus irregularis*. *New Phytologist* **220**(4): 1161-1171.

Chibucos MC, Soliman S, Gebremariam T, Lee H, Daugherty S, Orvis J, Shetty AC, Crabtree J, Hazen TH, Etienne KA. 2016. An integrated genomic and transcriptomic survey of mucormycosis-causing fungi. *Nature Communications* **7**(1): 1-11.

Corrochano LM, Kuo A, Marcet-Houben M, Polaino S, Salamov A, Villalobos-Escobedo JM, Grimwood J, Álvarez MI, Avalos J, Bauer D. 2016. Expansion of signal transduction pathways in fungi by extensive genome duplication. *Current Biology* **26**(12): 1577-1584.

Kämper J, Kahmann R, Bölker M, Ma L-J, Brefort T, Saville BJ, Banuett F, Kronstad JW, Gold SE, Müller O. 2006. Insights from the genome of the biotrophic fungal plant pathogen Ustilago maydis. *Nature* **444**(7115): 97-101.

Ma L-J, Ibrahim AS, Skory C, Grabherr MG, Burger G, Butler M, Elias M, Idnurm A, Lang BF, Sone T. 2009. Genomic analysis of the basal lineage fungus Rhizopus oryzae reveals a whole-genome duplication. *PLoS Genet* **5**(7): e1000549.

Martin F, Aerts A, Ahrén D, Brun A, Danchin E, Duchaussoy F, Gibon J, Kohler A, Lindquist E, Pereda V. 2008. The genome of *Laccaria bicolor* provides insights into mycorrhizal symbiosis. *Nature* **452**(7183): 88-92.

Martin F, Kohler A, Murat C, Balestrini R, Coutinho PM, Jaillon O, Montanini B, Morin E, Noel B, Percudani R. 2010. Périgord black truffle genome uncovers evolutionary origins and mechanisms of symbiosis. *Nature* **464**(7291): 1033-1038.

Mondo SJ, Dannebaum RO, Kuo RC, Louie KB, Bewick AJ, LaButti K, Haridas S, Kuo A, Salamov A, Ahrendt SR. 2017a. Widespread adenine N6-methylation of active genes in fungi. *Nature genetics* **49**(6): 964-968.

Mondo SJ, Lastovetsky OA, Gaspar ML, Schwardt NH, Barber CC, Riley R, Sun H, Grigoriev IV, Pawlowska TE. 2017b. Bacterial endosymbionts influence host sexuality and reveal reproductive genes of early divergent fungi. *Nature Communications* **8**(1): 1-9.

Montoliu-Nerin M, Sánchez-García M, Bergin C, Grabherr M, Ellis B, Kutschera VE, Kierczak M, Johannesson H, Rosling A. 2020. Building de novo reference genome assemblies of complex eukaryotic microorganisms from single nuclei. *Scientific Reports* **10**(1): 1-10.

Morin E, Miyauchi S, San Clemente H, Chen EC, Pelin A, de la Providencia I, Ndikumana S, Beaudet D, Hainaut M, Drula E. 2019. Comparative genomics of *Rhizophagus irregularis*, *R. cerebriforme*, *R. diaphanus* and

Gigaspora rosea highlights specific genetic features in Glomeromycotina. *New Phytologist* **222**(3): 1584-1598.

Pomraning KR, Bredeweg EL, Kerkhoven EJ, Barry K, Haridas S, Hundley H, LaButti K, Lipzen A, Yan M, Magnuson JK. 2018. Regulation of yeast-to-hyphae transition in Yarrowia lipolytica. *MSphere* **3**(6).

Schwartze VU, Winter S, Shelest E, Marcet-Houben M, Horn F, Wehner S, Linde J, Valiante V, Sammeth M, Riege K. 2014. Gene expansion shapes genome architecture in the human pathogen *Lichtheimia corymbifera*: an evolutionary genomics analysis in the ancient terrestrial mucorales (Mucoromycotina). *PLoS Genet* **10**(8): e1004496.

Schwessinger B, Sperschneider J, Cuddy WS, Garnica DP, Miller ME, Taylor JM, Dodds PN, Figueroa M, Park RF, Rathjen JP. 2018. A near-complete haplotype-phased genome of the dikaryotic wheat stripe rust fungus *Puccinia striiformis* f. sp. tritici reveals high interhaplotype diversity. *MBio* **9**(1).

Sun X, Chen W, Ivanov S, MacLean AM, Wight H, Ramaraj T, Mudge J, Harrison MJ, Fei Z. 2019. Genome and evolution of the arbuscular mycorrhizal fungus *Diversispora epigaea* (formerly *Glomus versiforme*) and its bacterial endosymbionts. *New Phytologist* **221**(3): 1556-1573.

Uehling J, Gryganskyi A, Hameed K, Tschaplinski T, Misztal P, Wu S, Desirò A, Vande Pol N, Du Z, Zienkiewicz A. 2017. Comparative genomics of *Mortierella elongata* and its bacterial endosymbiont *Mycoavidus cysteinexigens*. *Environmental Microbiology* **19**(8): 2964-2983.

Wang D, Wu R, Xu Y, Li M. 2013. Draft genome sequence of *Rhizopus chinensis* CCTCCM201021, used for brewing traditional Chinese alcoholic beverages. *Genome announcements* **1**(2).

Wood V, Gwilliam R, Rajandream M-A, Lyne M, Lyne R, Stewart A, Sgouros J, Peat N, Hayles J, Baker S. 2002. The genome sequence of *Schizosaccharomyces pombe*. *Nature* **415**(6874): 871-880.
